# Systemic responses in a tolerant olive (*Olea europaea* L.) cultivar upon root colonization by the vascular pathogen *Verticillium dahliae*

**DOI:** 10.3389/fmicb.2015.00928

**Published:** 2015-09-16

**Authors:** Carmen Gómez-Lama Cabanás, Elisabetta Schilirò, Antonio Valverde-Corredor, Jesús Mercado-Blanco

**Affiliations:** Department of Crop Protection, Institute for Sustainable Agriculture, Consejo Superior de Investigaciones CientíficasCórdoba, Spain

**Keywords:** verticillium wilt, *Olea europaea*, vascular pathogen, systemic defense responses, tolerance, susceptibility, defoliating pathotype

## Abstract

Verticillium wilt of olive (VWO) is caused by the vascular pathogen *Verticillium dahliae*. One of the best VWO management measures is the use of tolerant cultivars; however, our knowledge on VWO tolerance/resistance genetics is very limited. A transcriptomic analysis was conducted to (i) identify systemic defense responses induced/repressed in aerial tissues of the tolerant cultivar Frantoio upon root colonization by *V. dahliae*, and (ii) determine the expression pattern of selected defense genes in olive cultivars showing differential susceptibility to VWO. Two suppression subtractive hybridization cDNA libraries, enriched in up-regulated (FU) and down-regulated (FD) genes respectively, were generated from “Frantoio” aerial tissues. Results showed that broad systemic transcriptomic changes are taking place during *V. dahliae*-“Frantoio” interaction. A total of 585 FU and 381 FD unigenes were identified, many of them involved in defense response to (a)biotic stresses. Selected genes were then used to validate libraries and evaluate their temporal expression pattern in “Frantoio.” Four defense genes were analyzed in cultivars Changlot Real (tolerant) and Picual (susceptible). An association between *GRAS1* and *DRR2* gene expression patterns and susceptibility to VWO was observed, suggesting that these transcripts could be further evaluated as markers of the tolerance level of olive cultivars to *V. dahliae*.

## Introduction

Verticillium wilt of olive (*Olea europaea* L.) (VWO) is one of the most serious diseases affecting this relevant woody crop in many regions of the Mediterranean Basin. It is caused by the soil-borne fungus *Verticillium dahliae* Kleb. The disease is very difficult to control and the implementation of an integrated disease management strategy is therefore recommended. This framework must combine measures such as the use of tolerant cultivars, pathogen-free propagation material, appropriate cultural practices, and/or application of biological control agents (BCAs) (López-Escudero and Mercado-Blanco, 2011). Obviously, the use of olive varieties tolerant/resistant to VWO would be the most efficient and environmentally-friendly approach to control the disease (Tsror, 2011; Arias-Calderón et al., 2015). Up-to-date, however, no olive cultivar has been reported as fully resistant to VWO, although a number of studies have aimed to search and evaluate sources of genetic resistance to *V. dahliae* (López-Escudero and Mercado-Blanco, 2011, and references therein). We use the term tolerance (Robb, 2007) to refer to those olive cultivars able to cope with *V. dahliae* infections without developing severe symptoms of the disease (i.e., “Frantoio” and “Changlot Real”) in contrast to susceptible ones (i.e., “Picual”). “Frantoio” is considered one of the most tolerant cultivar to this harmful disease. A recent study has shown that “Frantoio” gave rise to a high number of tolerant seedlings, even when crossed with a very susceptible cultivar such as “Picual” (Trapero et al., 2015). However, not all VWO-tolerant genitors conferred tolerance to their offspring. For instance, “Changlot Real” and “Empeltre,” which are also considered very tolerant to *V. dahliae* (López-Escudero et al., 2004; Martos-Moreno et al., 2006; Trapero et al., 2013), mostly produced susceptible descendants (Trapero et al., 2015).

Understanding the mechanisms triggered in the host plant by the presence of the pathogen would be instrumental to design novel disease control strategies. Even though our knowledge on plant-pathogen interactions has been enhanced from studies based on powerful histological, histochemical, microscopy, molecular, and “omics” approaches, the information about the genetic bases underlying plant defense responses against vascular (Yadeta and Thomma, 2013) and/or root pathogens (Okubara and Paulitz, 2005; Larroque et al., 2013) is yet scant. Defense mechanisms deployed by the host plant can be the generation of structural barriers, i.e., tyloses (Dixon and Pegg, 1969), activation of metabolic responses, i.e., phytoalexins biosynthesis (Hammerschmidt, 1999), and/or mounting/triggering complex defense-related genetic cascades mediated by diverse signaling molecules, i.e., salicylate, jasmonate, etc. (Derksen et al., 2013). In the case of *V. dahliae* attacks, plant tissue responses so far reported can be structural, i.e., formation of tyloses in the xylem and/or biochemical, i.e., phenolic compounds accumulation (Baídez et al., 2007; Markakis et al., 2010). Moreover, these responses can be either constitutive (Mueller and Morgham, 1993) or induced in response to the pathogen infection (Daayf et al., 1997; Markakis et al., 2010). In olive, tolerance of cultivar Frantoio to *V. dahliae* has been proposed to be mediated by biochemical mechanisms activated in the root tissues rather than by plant structural characteristics such as vascular plugging (Bubici and Cirulli, 2012). These reactions are reported to be less noticeable in susceptible than in tolerant cultivars (Baídez et al., 2007; Markakis et al., 2010; Bubici and Cirulli, 2012). Interestingly, Daayf et al. (1997) observed similar responses (i.e., accumulation of paramural and cell wall coatings, phenolic compound deposits) in cotton (*Gossypium hirsutum* L.) plants infected by *V. dahliae*, these reactions being also more pronounced in tolerant than in susceptible cultivars. Up-to-date, no genetic and/or genomic information about systemic defense responses taking place in olive upon root colonization by *V. dahliae* is available, nor whether these responses could be related to VWO susceptibility level. Therefore, the objectives of this study were: (i) to elucidate whether early systemic responses can be differentially triggered in above-ground tissues of “Frantoio” plants upon root inoculation with a representative of the *V. dahliae* highly-virulent, defoliating (D) pathotype; and (ii) to determine whether specific systemic defense responses correlate with the differential VWO susceptibility level showed by diverse olive cultivars. From the wide range of differential responses found, seven genes identified in “Frantoio” cDNA libraries, namely transcription factor (TF) *GRAS1*, caffeoyl-O-methyltransferase (*CO-MT*), defensin protein 1 (*DEF*), disease resistance-responsive protein (*DRR2*), 1-aminocyclopropane-1-carboxylate oxidase (*ACO*), pathogenesis related protein 10 (*PR10*), and acetone cyanohydrin lyase (*ACL*) were selected for validation and to assess time-course gene expression pattern during *V. dahliae*-“Frantoio” interaction. We also analyzed the expression of *ACL, ACO, DRR2*, and *GRAS1* in two additional olive cultivars differing in VWO susceptibility to assess their potential as markers associated with tolerance to *V. dahliae* in a woody host of commercial relevance.

## Materials and methods

### Plant material and olive root inoculation with *Verticillium dahliae*

Two different olive-*V. dahliae* experiments were performed. In the first one, olive plants (3-month-old) of the tolerant cultivar Frantoio were utilized to generate cDNA libraries (see below). A second experiment, aiming to evaluate gene expression pattern of selected genes (see below), was carried out with olive cultivars Picual (VWO susceptible, 3-month-old), Frantoio and Changlot Real, (VWO tolerant, 8-month-old) (López-Escudero et al., 2004; Martos-Moreno et al., 2006). All olive plants originated from a commercial nursery located in Córdoba (southern Spain). Prior to *V. dahliae* treatment, plants were acclimated for 3 weeks in a growth chamber under conditions described below. Olive plants manipulation and root dip inoculation in a conidial suspension (30 min, 1·10^7^ conidia ml^−1^) of *V. dahliae* isolate V937I, representative of the highly-virulent D pathotype (Maldonado-González et al., 2015), were performed as previously described (Mercado-Blanco et al., 2002). Roots of control plants (non-inoculated) were dipped in water. Then, plants were individually transplanted into polypropylene pots containing an autoclaved sandy substrate (Prieto and Mercado-Blanco, 2008). Plants were maintained at controlled conditions (23 ± 1°C, 60–90% relative humidity, 14-h photoperiod of fluorescent light at 360 μE m^−2^) during 21 days. In order to ease plant stress after manipulation, inoculation and transplanting, the photoperiod was increased gradually (Gómez-Lama Cabanás et al., 2014). In the first experiment, aerial tissues (stems and leaves) of each olive plant were sampled at 8 h and 1, 2, 3, 4, 5, 6, 7, 10, 13, 15, and 21 (two plants/time point) DAI (days after inoculation) to obtain a broad range of differential (induced or repressed genes) responses. Therefore, aerial tissues of 48 plants (24 *V. dahliae* inoculated and 24 non-inoculated) were sampled, rapidly frozen in liquid nitrogen, and stored at −80°C until processing. In the second experiment, above-ground tissues (stems and leaves) of 24 plants (12 *V. dahliae* inoculated and 12 non-inoculated; two plants/time point/cultivar) were sampled at 2 and 10DAI. In addition, roots of three plants per cultivar were collected at 15DAI for assessment of *V. dahliae* infection. All samples were stored as described above.

### mRNA purification

Isolation of total RNA from olive tissue samples of the two experiments was performed according to Asif et al. (2000). RNA was treated with DNaseI (Roche Applied Science, Mannheim, Germany) according to the manufacturer's instructions. In the case of the first experiment (cDNA libraries generation), all RNA samples corresponding to each treatment (*V. dahliae*-inoculated and non-inoculated plants) were pooled separately to obtain two independent RNA pools prior to mRNA purification. Poly A+ mRNA was purified from approximately 400 μg of total RNA of each pool using the Dynabeads® mRNA Purification Kit (Dynal Biotech, Oslo, Norway) according to the manufacturer's indications. Purity and quality of all RNA samples were assessed electrophoretically and by spectrophotometry using a ND-1000 Spectrophotometer (NanoDrop Technologies, Wilmington, DE).

### Generation of “suppression substractive hybridization” cDNA libraries

Two different cDNA libraries were constructed from aerial olive tissues originated from the first experiment (“Frantoio”-*V. dahliae* V937I). “Suppression Subtractive Hybridization” (SSH) technology (Diatchenko et al., 1996) was used in order to clone and identify systemically FU library and FD library genes during the *V. dahliae*- “Frantoio” roots interaction. The PCR-Select™ cDNA Subtraction Kit (BD Biosciences, Palo Alto, CA) was used to generate cDNA libraries, following the manufacturer's instructions and as previously described (Schilirò et al., 2012; Gómez-Lama Cabanás et al., 2014). The constitutively-expressed β-actin gene from olive (Accession number AF545569), whose expression was checked as not influenced by *V. dahliae* inoculation, was used to check subtraction efficiency. The fragment (308-bp) was amplified using the primer pair Act1-fw: 5′-GCTTGCTTATGTTGCTCTCGAC-3′/Act1-rv: 5′-TGATTTCCTTGCTCATACGGTC-3′.

### Cloning and sequencing

cDNAs resulting from each SSH were ligated in the pGEM-T Easy Vector (Promega, Madison, WI) and cloned into *Escherichia coli* CH3-Blue competent cells (Bioline, London, UK) according to manufacturer's instructions. Positive (white) colonies were selected and picked in 96-well microtiter plates containing LB medium amended with 100 mg ml^−1^ ampicillin and then incubated at 37°C for 22 h. Finally, forward T7 universal primer was utilized to sequence 1344 bacterial clones from each SSH library. DNA sequencing was performed at a commercial service (Sistemas Genómicos S.L., Valencia, Spain).

### Bioinformatics analysis of ESTs

Contaminating vector and adaptors sequences were removed from ESTs (Expressed Sequence Tags) by mass alignment using the “CLC Main Workbench 6.8.1” (CLC bio, Aarhus, Denmark) software. Sequences that showed low quality or length (<100 bp) were ruled out from the analysis. EST data set assembly was performed by using the “CLC Main Workbench 6.8.1,” aiming to find contiguous sequences and redundancy. Computational annotation of ESTs originating from *V. dahliae*-“Frantoio” interaction was carried out by using the software “Blast2GO PRO trial” (Conesa et al., 2005) available at https://www.blast2go.com/. To find homologies, the non-redundant (nr) GenBank protein database was searched, running the Blastx algorithm (Altschul et al., 1990) with the *E*-value set to 1.0E-3 and the High-scoring Segment Pairs (HSP) length cut off fixed to 33 (Schilirò et al., 2012). The ‘Blast2GO PRO trial software was used to perform Gene Ontology (GO) analysis from retrieved database matches. InterPro Scan (Zdobnov and Apweiler, 2001) and functional annotation was used to associate functional information and GO terms to the protein of interest, implementing the specific tool in the Blast2GO software with the default parameters. The “Augment Annotation by ANNEX” function was finally used to improve the annotation profiles information. The GOslim “goslim_plant.obo” was run to achieve specific plant GO terms by means of a plant-specific reduced version of the GO (available at http://www.arabidopsis.org/). Enzyme mapping of annotated sequences was retrieved by direct GO to Enzyme annotation and used to query the Kyoto Encyclopaedia of Genes and Genomes (KEGG - http://www.genome.jp/kegg/) to define the main metabolic pathways involved. The distribution of hits obtained against entries for other plants within the NCBI database was used to get a descriptive view of the newly generated dataset.

### Accession numbers

ESTs reported in this study have been deposited in the dbESTs database of the National Center for Biotechnology Information (NCBI) under GenBank accession numbers JZ844237 (dbEST_Id79672628)-JZ844946 (dbEST_Id79673337) (FU library) and JZ822972 (dbEST_Id79640674)-JZ823534 (dbEST_Id79641236) (FD library).

### Data validation and time-course gene expression profiles during *Verticillium dahliae*-olive “frantoio” interaction

Selected ESTs identified in FU and/or FD cDNA libraries by the Blast2GO tool were used for validation by quantitative real-time PCR (qRT-PCR) experiments. Seven transcripts from the whole dataset of nr sequences, two of them from FU (*GRAS1* [Accession number JZ844283] and *CO-MT* [JZ844695]), four from FD (*DEF* [JZ823187], *DRR2* [JZ823156], *ACO* [JZ823262] and *PR10* [JZ823324]), and one identified in both FU and FD (salicylic acid-binding protein 2-like = acetone cyanohydrin lyase, *ACL* [JZ844792 and JZ823163]), all involved in plant defense responses, were chosen for data validation (Table 1). All these ESTs fulfilled the criteria of >100-bp long and *E* = 1.0E-3. Expression patterns of these genes in above-ground organs were assessed at 8 h, 2, 4, 10, and 15DAI after *V. dahliae* root inoculation. *ACL* and *CO-MT* primer pairs have been previously described (Schilirò et al., 2012). The “CLC Main Workbench 6.8.1” (CLC bio) software was used to design specific primer pairs for the remaining genes (Table 1). All primer pairs were empirically tested for their specificity in the temperature range 53°C–63°C by conventional PCR. To find the pertinent concentration range at which target cDNA amplified more efficiently, specific qRT-PCR experiments were implemented using cDNAs synthesized from 10-fold serially diluted (1 μg, 100 ng, 10 ng, 1 ng, 100 pg) RNA samples. To draw standard curves for each selected transcript reverse transcribed cDNA was used from serial dilutions (300, 30, 3, 0.3 ng) of remnant total RNA samples not used for SSH. Ct values and the log cDNA concentrations were linearly correlated for each of the examined genes, and PCR efficiencies were automatically calculated by iQ5 optical system software v.2.1 (BioRad, Hercules, CA). Synthesis of cDNA was performed from 100 ng of total RNA in each of five different times assayed using the “iScript cDNA Synthesis Kit” (BioRad, Hercules, CA) following the manufacturer's indications. qRT-PCR experiments and analyses were done in a thermal cycler iQ5 Real-Time PCR System (BioRad) provided with a 96-well sample block. Relative expression for each selected gene was repeated at least once in independent qRT-PCR assays, and three replicas per condition studied and per plate were routinely included. All qRT-PCR reactions, melting profiles and normalizations were performed as previously described (Gómez-Lama Cabanás et al., 2014). Relative expression levels at different times were calculated according to Livak and Schmittgen (2001). The average of each expression gene fold change was categorized as follows: “low” ≥ −1.0 to ≤ 1.0; “medium” ≥ −2.0 to < −1.0 or > 1.0 to ≤ 2.0; and “high,” < −2.0 or > 2.0 (Kim et al., 2008). All relative expression data in four different times for each gene were represented as means ± SD of at least two independent qRT-PCR experiments, each performed with triplicate samples.

**Table 1:** **List of selected transcripts induced in “Frantoio” aerial tissues upon ***Verticillium dahliae*** colonization used in qRT-PCR experiments**.

**Clone ID**	**Putative gene**	**Process**	**Primer pair**	**Amplicon length (bp)**	**Linear equation**	***R*^2^**	**PCR efficiency**
FD07-B04T7	1-Aminocyclopropane-1-carboxylate oxidase	Ethylene biosynthesis	Fw: CTCAAGTTGATCCCCAAT Rv: GCATTCCATGGCTCTAAA	231	y = −3.334*x* + 26.333	0.99	99.5
FD04-H01T7	Defensin protein 1	Defense response	Fw:ACACCATGAGCAGGAAAA Rv:TGGCTATTGCAGGGGATT	166	y = −3.452*x* + 27.980	0,99	94.8
FU08-D05T7	Caffeoyl-o-methyltransferase	Phenylpropanoids biosynthesis	Fw:ACCAGAGGCCATGAAAGAAC Rv:ATTGCCAAAATCTTCCCATC	204	y = −3.399*x* + 26.905	1.00	96.9
FD08-H06 T7	Pathogenesis-related protein 10	Defense response	Fw:GATGTGTGGAGAGGCTTT Rv: CGTCATTTTTCTTCCTAGGT	153	y = −3.351*x* + 23.514	0.99	98.8
FD-C16 FU-C145	Acetone-cyanohydrin lyase	Salicylic acid-binding protein 2	Fw: GAAAGAGATGGAAGCGGAAA Rv: ACACAGGGAAATGCATCAAA	246	y = −3.390*x* + 28.218	0.99	97.2
FU01-E11T7	Transcription factor GRAS1	Signal transduction defense response	Fw:TCAGTGGGTGTTCCTTATT Rv: GGTGCAGCATATAAGGAAA	269	y = −3.415*x* + 31.094	0.99	96.3
FD-C65	Disease resistance-responsive family protein	Defense response	Fw:CCAATGCCCGTAAAGTAA Rv: TACAGCGTTTCTTCCCAA	311	y = −3.774*x* + 26.080	1.00	84.1
(1) AF545569	*Olea europea* beta-actin (act1)	Citoskeletal integrity	Fw: GCTTGCTTATGTTGCTCTCGAC Rv: TGATTTCCTTGCTCATACGGTC	308	y = −3.465*x* + 27.632	0.99	94.4

*For all transcripts, relative expression analysis was repeated at least two times in independent qRT-PCR experiments (see text and **Figure 2**). Clone ID, gene name, biological process, primers sequences, amplicon length, linear equations, correlation coefficients (R^2^), and PCR efficiencies are indicated. Fw, Forward; Rv, Reverse; FU, Frantoio aerial tissues induced gene; FD, Frantoio aerial tissues repressed gene; (1), olive act-1 gene used as reference to normalize relative expression (Gene Bank Accession Number)*.

### Gene expression patterns in olive cultivars displaying differential *Verticillium dahliae* susceptibility level

Four genes were selected to assess their expression patterns at two different time points (2 and 10DAI) in olive cultivars “Picual” (susceptible) and “Frantoio” and “Changlot Real” (tolerant) (Trapero et al., 2013) from the second experiment. The expression of one up-regulated (*GRAS1*), two down-regulated (*ACO* and *DRR2*) and one found in both libraries (*ACL*) genes was studied following the approach previously described. Linear equations, correlation coefficients (R^2^) and PCR efficiencies were estimated for each transcript (Table 2). For each selected gene, expression was quantified at least two times in independent qRT-PCR experiments, and three replicas per time point studied and per plate were routinely included.

**Table 2:** **List of selected transcripts induced in olive aerial tissues upon ***Verticillium dahliae*** colonization used in qRT-PCR experiments with susceptible (Picual) and tolerant (Frantoio and Changlot Real) olive cultivars**.

**Putative gene**	**Cultivars**								
	**Picual**			**Frantoio**			**Changlot real**		
	**Linear equation**	**R^2^**	**PCR efficiency**	**Linear equation**	**R^2^**	**PCR efficiency**	**Linear equation**	**R^2^**	**PCR efficiency**
1-Aminocyclopropane-1-carboxylate oxidase	y = −3.580*x* + 26.556	0.99	90.2	*y* = −3.305*x* + 27.123	0.98	100.7	y = −3.542*x* + 29.629	0.99	91.6
Acetone-cyanohydrin lyase	y = −3.311*x* + 28.941	0.99	100.5	y = −3.447*x* + 28.292	0.99	95.0	y = −3.536*x* + 31.503	0.98	91.8
Transcription factor GRAS1	y = −3.275*x* + 27.722	0.99	102.0	y = −3.301*x* + 28.952	0.99	100.9	y = −3.341*x* + 29.082	0.99	99.2
Disease resistance-responsive family protein	y = −3.753*x* + 24.626	0.99	84.7	y = −3.808*x* + 25.865	0.99	99.4	y = −3.528*x* + 27.767	0.98	92.1
*Olea europea* beta-actin (act1)	y = −3.491*x* + 28.287	0.99	93.4	y = −3.475*x* + 27.558	0.98	94.0	y = −3.718*x* + 28.072	0.99	85.8

*For all transcripts, relative expression (RE) analysis was repeated at least two times in independent qRT-PCR experiments (see text and **Figure 3**). Gene name, linear equations, correlation coefficients (R^2^) and PCR efficiencies are indicated for each gene/cultivar combination*.

### Detection of *V. dahliae* in root tissues

Presence of *V. dahliae* in olive roots was checked by PCR. Reactions were performed containing 25 ng of DNA, 0.1 μM of *V. dahliae* specific primers (DB19 and DB22) (Carder et al., 1994), 2x iQ™ SYBR® Green Supermix (BioRad) and H_2_O up to a total volume of 20 μL. PCR protocol was: denaturation for 4 min at 95°C, followed by 50 cycles of 1 min at 94°C, 45 s at 54°C and 45 s at 72°C and a final extension step of 10 min at 72°C. Melting curves of products were assessed from 54°C to 100°C to confirm the amplification of single PCR bands. For all samples reaction protocol was as follows: 5 min at 95°C for initial denaturation, cooling to 54°C and melting from 54°C to 100°C with a 0.5°C transition rate every 10 s. For each plant root, presence of *V. dahliae* DNA was verified two times in independent experiments, and three replicas per point studied and per plate were routinely included.

## Results

### Construction of cDNA libraries enriched in differentially-expressed olive genes from aerial tissues during the colonization of “frantoio” roots by *V. dahliae*

Two different cDNA libraries enriched in up- and down-regulated transcripts from above-ground tissues of olive cv. Frantoio were generated. A total of 1344 EST sequences were sequenced and analyzed for each library. On the one hand, 585 unigenes were identified in the up-regulated cDNA library (FU), which were assembled into 57 contigs (average length 524 bp) each composed of 2–4 sequences and 528 singlets (average length 427 bp) (Table S1). On the other hand, ESTs from the down-regulated cDNA library (FD) were eventually assembled into 52 contigs (average length 479 bp), each composed of 2–13 sequences, and 329 singlets (average length 382 bp) (Table S2). Despite the fact that the number of ESTs sequenced in both libraries was the same, the number of up-regulated unigenes found in aerial tissues was considerably higher than that of down-regulated unigenes.

Querying (Blastx) the nr NCBI database allowed the attribution of homologous hits for nearly 77% of the ESTs in both libraries. Hits distribution of the complete EST set against sequences from different plant species revealed that 39% of the ESTs matched to coding sequences previously identified in genomes of woody plants such as robusta coffee (*Coffea canephora* L. Linden) (9.8% present in FU and 7.1% in FD) grapevine (*Vitis vinifera* L.,) (6.1 and 6.5%), and cacao (*Theobroma cacao* L.) (2.7 and 2%). *E*-values ranged from 1.00E-03 to 5.54E-169 for FU cDNA library and 1.66E-3 to 1.94E-162 for FD cDNA library. Only 3.5 and 5.9% of the unigenes found in FU and FD libraries, respectively, showed significant homology with olive genes [NCBIdbEST (http://www.ncbi.nlm.nih.gov/dbEST/)] (Tables 3, 4). This indicates an as yet important lack of genetic/genomic information for this relevant woody crop. Finally, 23% of induced and repressed transcripts found in both libraries were of unknown function.

**Table 3:** **List of induced EST sequences identified after Blastx analysis as homologous to olive genes previously indexed in databases**.

**EST sequence name**	**Putative protein function**	**Accession number**	***E*-Value**
^*^FU02-D07T7	Beta-glucosidase 12-like	AAL93619	1.89E-41
FU02-E10T7	Lipoxygenase family protein	ACD43483	1.81E-85
FU03-C10T7	Thaumatin-like protein	E3SU11	5.49E-28
FU04-D05T7	Alcohol dehydrogenase	AEQ04839	2.49E-91
FU04-G10T7	24-methylenesterol c-methyltransferase 2	AGR55393	1.81E-74
FU05-E01T7	Lipid transfer protein	ABS72013	6.81E-19
FU06-C04T7	Beta-glucosidase 12-like	AAL93619	1.49E-41
FU08-D04T7	Serine mitochondrial-like	ABS72016	7.01E-136
FU11-A09T7	b chain structures of alkaloid biosynthetic glucosidases decode substrate specificity	AAL93619	7.38E-44
FU11-B02T7	Beta-glucosidase 44-like	AAL93619	1.19E-18
FU11-F10T7	Ribulose- -bisphosphate carboxylase oxygenase activase	ABS72022	5.60E-90
FU12-A03T7	Cytochrome b5-like	CAA04702	5.53E-14
FU12-F09T7	Beta-glucosidase isozyme 2 precursor	AAL93619	8.19E-48
FU13-D06T7	Cytochrome p450 subunit cyp72a13	AFS28694	1.38E-21
FU14-H02T7	Carbonic chloroplast precursor	CBL86547	2.77E-48
FU-C17	Hypothetical protein, partial	AFP49328	9.00E-13
FU-C58	Hypothetical protein, partial	AFP49328	1.01E-05
FU- C224	Linoleate 13s-lipoxygenase 2- chloroplastic-like	ACD43485	3.09E-116

* *Unigene found in both libraries with the same accession number*.

**Table 4:** **List of repressed EST sequences identified after Blastx analysis as homologous to olive genes previously indexed in databases**.

**EST sequence name**	**Putative protein function**	**Accession number**	***E*-Value**
FD01-E11T7	Protein chloroplastic	AFP49328	1.69E-42
FD01-H08T7	Ole e 5 olive pollen allergen	ABX26138	1.13E-45
FD02-E02T7	Chloroplast ribulose—bisphosphate carboxylase oxygenase small subunit	ABS71998	3.00E-08
FD03-C06T7	Glycolate oxidase	ABS72011	1.40E-67
FD03-E06T7	Photosystem i reaction center subunit chloroplastic-like	ABU39903	3.59E-48
FD03-F07T7	Fatty acid hydroperoxide lyase	ACD43482	6.90E-82
FD03-F09T7	4-hydroxy-3-methylbut-2-enyl diphosphate partial	AFS28680	3.21E-57
FD04-C07T7	Isopentenyl diphosphate isomerase	AFS28681	1.99E-68
FD04-H01T7	Defensin ec-amp-d2-like	ABS72000	1.66E-01
FD05-D05T7	Aquaporin tip1-3-like	ABB76813	2.30E-83
FD06-E12T7	Isopentenyl-diphosphate delta-isomerase i	ACF05532	4.17E-10
FD07-B09T7	Cytochrome p450 family protein	AFS28690	8.18E-66
FD07-E06T7	Hypothetical protein, partial	AFP49328	4.22E-06
FD08-F04T7	Ribulose-bisphosphate carboxylase oxygenase activase	ABS72022	2.25E-91
FD09-A03T7	Glyceraldehyde-3-phosphate dehydrogenase chloroplastic	ABS72003	3.44E-50
FD14-E01T7	Achain crystal structure of perakine founder member of a novel akr subfamily with unique conformational changes during nadph binding	ABS72001	4.39E-27
FD-C129	Thaumatin-like protein	E3SU11	9.31E-49
FD-C156	Metallothionein 1	AFP49330	5.50E-15
FD-C163	Salt tolerance-related protein	ABS72020	4.80E-62
^*^FD-C203	Beta-glucosidase partial protein	AAL93619	1.78E-111

* *Unigene found in both libraries with the same accession number*.

### Functional characterization of EST data set

**Figure 1:**
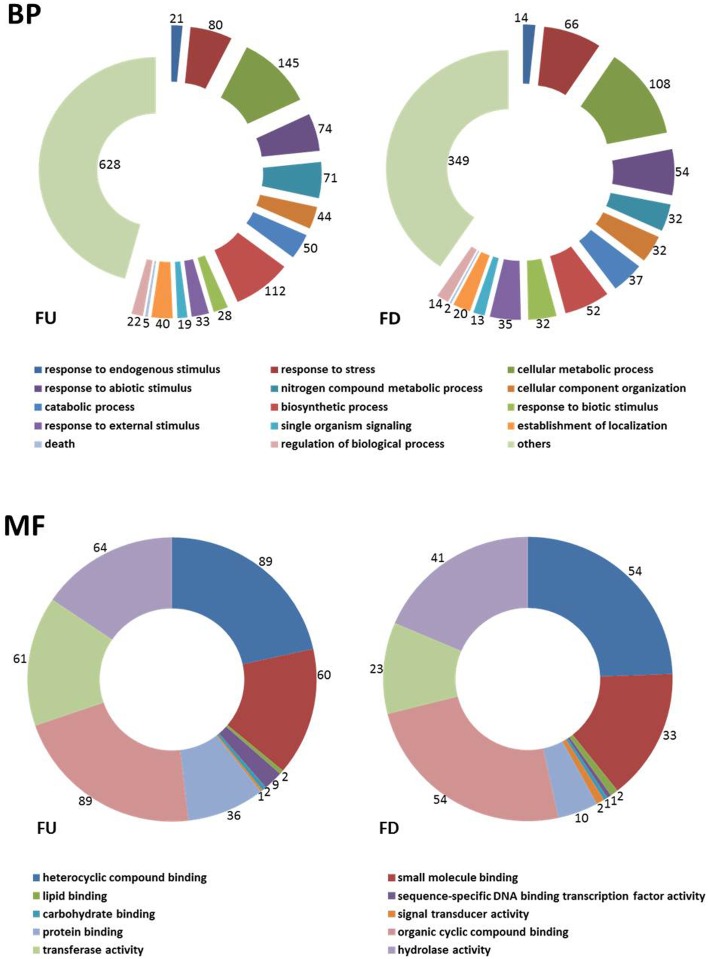
**“Level 3” Gene Ontology (GO) “Biological processes” (BP) and “Molecular function” (MF) terms distribution of 381 unigenes repressed (FD) and 585 induced (FU) in olive (***Olea europaea*** L.) aerial tissues colonized by ***Verticillium dahliae*****. Unigenes were categorized using the “Blast2GO” software.

Blast2GO analysis of the EST set enabled annotation of expressed sequences according to the terms of the three main GO vocabularies, i.e., “Biological Process” (BP), “Molecular Function” (MF), and “Cellular Component” (CC). GO annotation was only feasible for 57.3% (up-regulated genes) and 68.3% (down-regulated genes) of the sequences, i.e., 141 and 89 ESTs, respectively. A total of 140 FD and 88 FU assigned to “unknown” category and 1 FU and 1 FD assigned to “predicted” category were automatically excluded from the analysis by the program. Since a number of transcripts were identified by different GO terms, the mapped ESTs distribution for BP and MF main categories shown in Figure 1 resulted in more than 585 (FU) or 381 (FD) sequences. The distribution of assignments into GO categories “level 3” was 336 (FU) and 226 (FD) (for BP), 311 (FU) and 202 (FD) (for MF), and 266 (FU) and 201 (FD) (for CC). Regarding to BP main GO vocabulary, transcripts representing GO terms categories non-related to plant defense processes (e.g., anatomical structure development, primary metabolic process, reproductive process, etc.), were grouped as “other” (Figure 1, BP). Concerning to plant defense-related categories, ESTs found to be induced or repressed in above-ground organs upon *V. dahliae* olive root colonization were assigned to processes such as “response to stress” (80 FU and 66 FD unigenes), “response to abiotic stimuli” (74 FU and 54 FD), “response to biotic stimuli” (28 FU and 32 FD), “response to external stimuli” (33 FU and 35 FD) or “response to endogenous stimuli” (21 FU and 11 FD). GO terms included catalases (CATs), proteins involved in the phenylpropanoid pathway, ET biosynthesis (ACO) or terpenoids biosynthesis, proteins related to SA (ACL), linolenic acid metabolism and PR protein. In addition, we identified several transcription factors (TF) such as WRKY's (WRKY20, WRKY44, WRKY33, WRKY40) or GRAS1, ET-responsive TF rap2-12-like, elongation factors (EF-1α, EF-1β, and EF-1Δ) and proteins directly related with stress responses (for a complete list of unigenes, see Tables S1, S2).

For the main GO vocabulary term MF, 10 different categories were identified for “level 3” (Figure 1, MF). The three main categories were “heterocyclic compound binding” (89 unigenes in FU like disease resistance protein and 54 unigenes in FD such as ribulose-bisphosphate carboxylase oxygenase activase), “organic cyclic compound binding” (89 unigenes in FU as adp-ribosylation factor 2-like and 54 unigenes in FD such as glycine dehydrogenase) and “hydrolase activity” (64 unigenes in FU such as thaumatin-like protein and 41 unigenes in FD like cysteine proteinase inhibitor) (Figure 1 MF and Tables S1, S2).

Finally, the same pattern of categories distribution for main GO term vocabulary CC was found in both libraries (data not shown). Thus, most of the unigenes identified were assigned to: “cell part” (242 unigenes in FU such as glutamine synthetase and 187 unigenes in FD like selenium-binding protein 2) and “membrane-bounded organelle” (180 unigenes in FU like ET-responsive transcription factor rap2-12-like and 149 unigenes in FD as protein thylakoid chloroplastic-like) (Tables S1, S2).

### Identification of defense response genes in FU and FD libraries

Analysis of the 585 olive ESTs from the FU library showed that 19.8% of the induced genes in aerial olive tissues related with plant responses to different stimuli and stresses ([a]biotic, endogenous, extracellular, and/or external). For instance, genes potentially coding for a catalase or a tyrosyl-dna phosphodiesterase 1-like (response to stress), calcium-dependent protein kinase sk5-like or calmodulin-binding family protein (response to endogenous stimuli), auxin-responsive protein iaa14-like or cysteine proteinase 15a-like (response to external stimuli), ATP sulfurylase chloroplastic-like or 2-oxoisovalerate dehydrogenase subunit beta mitochondrial-like (response to extracellular stimuli), peptidyl-prolyl cis-trans isomerase fkbp65-like (response to wounding), gdt1-like protein 4, ET-responsive transcription factor rap2-12-like, or protein dehydration-induced 19 homolog 4-like (response to abiotic stimulus), ACL, pectin methylesterase, linoleate 13s LOX 2-chloroplastic-like, feroina like protein or thaumatin like-protein (response to biotic stimulus) and hva22-like protein a-like (response to abiotic and endogenous stimulus, response to stress), were found to be up-regulated. In addition, several TF related to plant defense responses (i.e., WRKY factors or GRAS1) were identified in the FU cDNA library as well (Table S1).

Similarly, analysis of the 381 olive ESTs from the FD library showed that 25.7% of the repressed genes in above-ground tissues were also related with plant responses to different stimuli and stresses. Thus, presence of the pathogen in the roots repressed genes related to response to biotic stimuli such as defensin, chloroplastic 6-phosphogluconolactonase and ribose-5-phosphate isomerase, oxoglutarate 3-dioxygenase-like or phosphatase 2c 25. Some unigenes were identified in both libraries coding for calcium (Ca^2+^)-binding proteins likely related with plant defense reaction, e.g., calmodulin, calmodulin binding, Ca^2+^ transporting ATPase, Ca^2+^ exchanger 4-like, and Ca^2+^ dependent protein kinase sk5-like, ACL, as well as β-amylases (chloroplast beta amylase isoform and inactive beta amylase 9-like) (Tables S1, S2). It is worth mentioning that some of these unigenes shared the putative protein function but had different sequences and accession numbers in the databases (Tables S1, S2), while others shared the putative protein function and accession number but sequences were different (e.g., glucosidases) (Tables 3, 4). We also detected 37 unigenes with the same sequence, putative protein function and accession number in both libraries, accounting for 7.2% (FU) and 9.7% (FD) of the sequences in the libraries. Finally, it is interesting to emphasize that GO term assignment revealed that 4% (FU library) and 19% (FD library) of the identified unigenes related with the photosynthesis process. The complete lists of ESTs induced and repressed in above-ground organs after inoculation of olive roots with *V. dahliae* are shown in Tables S1, S2, respectively. In addition, Tables S3, S4 display contigs identified in FU and FD cDNA libraries with their corresponding contiguous/overlapping ESTs. The 37 ESTs founded in both libraries and ESTs whose functions were unknown are not included in these tables.

### Data validation and time-course of expression of selected defense response olive genes to *Verticillium dahliae*

**Figure 2:**
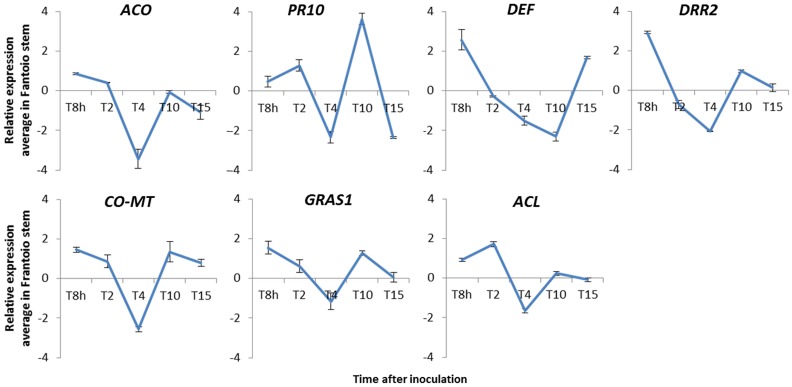
**Relative expression (RE) average of seven genes identified in FD and/or FU cDNA libraries from aerial tissues of “Frantoio” olive plants at different time points after ***Verticillium dahliae*** inoculation in roots**. *ACO*, 1-aminocyclopropane-1-carboxylate oxidase; *DRR2*, disease resistant response protein; *ACL*, acetone cyanohydrin lyase; *GRAS1*, transcription factor GRAS1; *CO-MT*, caffeoyl-o-methyltransferase; *PR10*, pathogenesis-related protein 10; and *DEF*, defensin protein 1. Error bars represent the SD from at least two independent qRT-PCR experiments. RE values (log2-fold-change values) were calculated according to the 2^−ΔΔ^^*Ct*^ method (Livak and Schmittgen, 2001).

Seven genes identified in FU, FD or both libraries, *CO-MT, GRAS1* (up-regulated genes), *ACO, PR10, DEF, DRR2* (down-regulated genes), and *ACL* (found in both libraries) were selected to analyze short- and mid-term (8 h, 2, 4, 10, and 15 DAI) gene expression pattern. Overall, results showed a gene repression at 4DAI, followed by an increase at 10DAI. Most of the genes reached the maximal relative expression level within the first hours after pathogen inoculation (Figure 2). The two up-regulated genes identified in the FU library (*CO-MT* and *GRAS1*) were validated at all-time points except at 4DAI. Down regulation of two (*ACO* and *DEF*) out of four genes identified in the FD library was confirmed at three different times after pathogen inoculation. On the contrary, down-regulated expression of *DRR2* and *PR10* was validated only at two times (Figure 2). Finally, *ACL* gene, found in both libraries, displayed an ambiguous expression pattern along time. The vast majority of relative fold changes were assigned to medium (>+1.0 to ≤ +2.0 or < −1.0 to ≥ −2.0) or low (≥ −1.0 to ≤ +1.0 or ≤ 1.0 to ≥ −1.0) categories of differential expression, but for some cases such as *DRR2* (8 h and 4DAI) and *DEF* (8 h and 10DAI), *ACO* and *CO-MT* (4DAI) or *PR10* (4, 10, and 15DAI) which were assigned to the high category (>+2 or < −2) (Figure 2). Linear equations, correlation coefficients (*R*^2^) and PCR efficiencies for each gene are shown in Table 1.

### Gene expression pattern of *GRAS1, ACO, DRR2* and *ACL* genes in susceptible and tolerant olive cultivars

**Figure 3:**
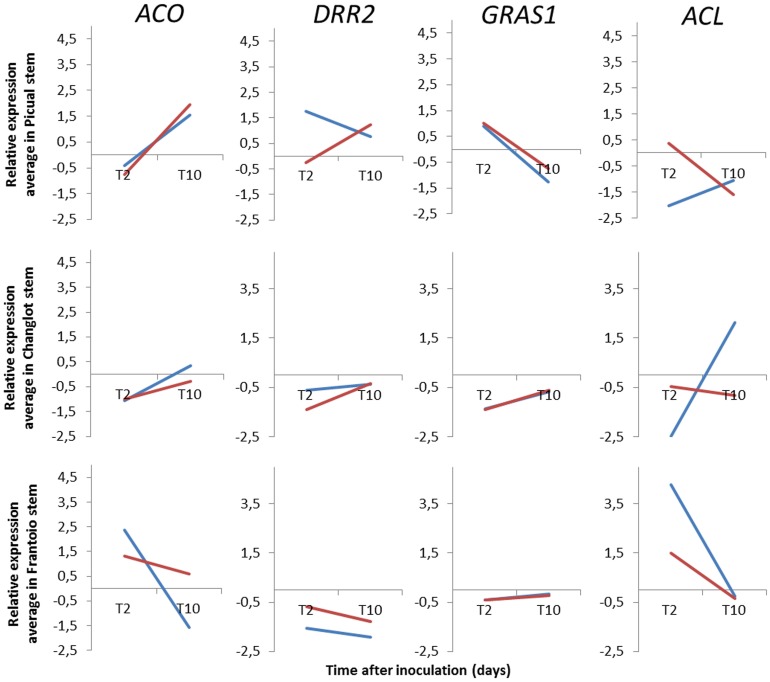
**Relative expression (RE) average of four genes from aerial tissues of inoculated tolerant (“Frantoio” and “Changlot Real”) and susceptible (“Picual”) olive cultivars at two different time points after ***Verticillium dahliae*** inoculation in roots**. *ACO*, 1-aminocyclopropane-1-carboxylate oxidase; *DRR2*, disease resistant response protein; *ACL*, acetone cyanohydrin lyase; *GRAS1*, transcription factor GRAS1; Relative expression values (log2-fold-change values) were calculated according to the 2^−ΔΔ^^*Ct*^ method (Livak and Schmittgen, 2001). Red and blue lines show RE scored for two different plants sampled at the indicated times after inoculation with the pathogen.

In order to compare the gene expression pattern of specific genes in different olive cultivars (“Picual,” susceptible to VWO, and “Frantoio” and “Changlot Real,” tolerant to VWO), four genes (*GRAS1, ACO, DRR2*, and *ACL*) were selected (Table 2). Their expression patterns were assessed at 2 and 10DAI. Relative expression for each gene/cultivar combination is shown in Figure 3 (see Table S5 for additional data). Time-course expression of each gene differed among cultivars but the two biological replicas (plants) tested by cultivar did not always show the same expression pattern. *ACO* expression displayed the same trend in “Picual” and “Changlot Real” in contrast to “Frantoio.” Indeed, this gene showed an induction trend from 2 to 10DAI, particularly in “Picual” plants, whereas it showed a trend to be repressed in “Frantoio” plants along this interval. *DRR2* expression is down-regulated in tolerant cultivars but up-regulated in the susceptible “Picual,” the two plants of this cultivar showing discrepant *DRR2* expression patterns though (Figure 3). Expression of *GRAS1* yielded the most consistent results, this gene being down regulated in tolerant cultivars at both sampling times. On the contrary, “Picual” plants showed a sharp fall in *GRAS1* expression levels along time. Finally, results for *ACL* gene expression were the most contentious since plants of the same cultivar (i.e., “Picual” and “Changlot Real”) showed opposite expression patterns. In contrast, the two biological replicas of “Frantoio” displayed a consistent down regulation trend from 2 to 10DAI. For this gene, however, no clear association between VWO susceptibility and expression of defense genes was found.

### Detection of *V. dahliae* DNA in roots of inoculated olive plants

Presence of *V. dahliae* was confirmed in inoculated plants of both tolerant (“Frantoio” and “Changlot Real”) and susceptible (“Picual”) plants. PCR assays allowed the detection of pathogen's DNA in roots of all inoculated plants. Control (non-inoculated) plants yielded negative results for *V. dahliae* DNA detection (data not shown).

## Discussion

Understanding the mechanisms conferring olive tolerance to *V. dahliae* could provide valuable information for (i) breeding new VWO-tolerant cultivars, (ii) developing novel pathogen diagnostic tools, and (iii) improving integrated management strategies for VWO control. By generating two cDNA libraries, enriched in up- and down-regulated genes respectively, we have explored for the first time the transcriptomic changes taking place in a commercially-relevant woody plant such as olive upon infection by the D pathotype of *V. dahliae*, the most lethal for olive cultivation. The tolerant olive cultivar Frantoio was selected for such primary aim. We focused on the identification of systemic responses taking place in aerial tissues, particularly those related to defense to different (a)biotic stresses. A secondary objective was to assess the expression pattern of selected genes involved in such responses in olive cultivars showing differential susceptibility to VWO. This could provide valuable information on genetic markers associated to *V. dahliae* tolerance/susceptibility posing relevant practical implications in VWO management.

Our study has shown that, upon *V. dahliae* root inoculation, a range of early plant defense responses to (a)biotic stresses are induced (i.e., *CO-MT*., *Hva22*) and repressed (i.e., *defensin, raffinose synthase*) at distant tissues in “Frantoio” plants. Besides, different classes of TF-coding genes involved in (a)biotic stresses (Archana et al., 2009) such as *GRAS1* and *WRKYs* (i.e., *WRKY44, WRKY33*, and *WRKY20*) were shown to be systemically up-regulated. For instance, Arabidopsis *WRKY33* plays an important role in resistance to necrotrophic pathogens (Zheng et al., 2006), while *A. thaliana WRKY20* has been involved in increased resistance to *Bacillus amyloliquefaciens* (Kjellin, 2012). Homologous genes to both *WRKYs* have been identified as systemically up-regulated in “Frantoio” upon root colonization by *V. dahliae*, suggesting the involvement of these TF in defense against a vascular pathogen. On the other hand, different elongation factors such as EF-1α were found in both cDNA libraries. The involvement of EF-1α in translation regulation during abiotic stresses in soybean (Chung et al., 2009), or during cold stress-related expression in barley and maize (Dunn et al., 1993; Berberich et al., 1995) has been reported. Finally, genes coding for β-amylases were also found in both libraries. These enzymes have been recently proposed as negative regulators in Arabidopsis partial resistance against *V. dahliae* (Gkizi et al., 2015). The role of detected olive elongation factors and β-amylases in the interaction olive-*V. dahliae* remains to be elucidated.

Seven genes related to defense responses (namely *ACO, CO-MT, ACL, GRAS1, DRR2, PR10*, and *DEF*) were selected to analyze their time-course expression patterns in “Frantoio.” ACO (ACC oxidase), involved in ethylene biosynthesis is regulated by a number of environmental factors and (a)biotic stresses (Díaz et al., 2002; Wang et al., 2002; Iwai et al., 2006). Furthermore, silencing of *ACO* gene was proven to affect the susceptible plant response to pathogen attack (Shan and Goodwin, 2006). CO-MT is involved in the phenylpropanoids pathway which plays important roles in plant development and (a)biotic stress responses (Dixon et al., 2002; Korkina, 2007). ACL is a salicylic acid binding protein involved in cyanogenic glycosides catabolism (Trummler and Wajant, 1997), related to chemical defense systems in plants under different biotic stresses (Ganjewala et al., 2010). GRAS1 is a TF belonging to a large protein family, many of them involved in plant response to (a)biotic stress (Mayrose et al., 2006; Sun et al., 2012). DRR2 is a disease resistance response protein of the dirigent family protein (DIR). Many DIR gene homologs have been detected in various plant species (Davin and Lewis, 2000). PR10 is a pathogenesis-related (PR) protein, a well-known group that constitute a defense response system under (a)biotic stresses (van Loon et al., 2006). Finally, DEF is a protein belonging to a plant defensin family proteins implicated in the first-line host defense against fungal pathogens (Thomma et al., 2002), although certain defensin genes are down-regulated (Moreno et al., 1994; Segura et al., 1998). Results from qRT-PCR revealed an overall decrease of the relative expression in all tested genes at 4DAI. This transient down-regulation was also observed in above-ground tissues during the interaction *Pseudomonas fluorescens* PICF7-olive (cv. Arbequina) roots (Gómez-Lama Cabanás et al., 2014). One possible explanation could be that plants undergo an overall gene expression repression at this time point, regardless whether they interact with a beneficial endophyte (Gómez-Lama Cabanás et al., 2014) or with a vascular pathogen (this study). Another explanation could be that plants were not exposed to a full day-light period during the first days of the experiment to avoid additional stress after manipulation. It is known that light exposure and intensity can influence the ability of plants to defend themselves from biotic stresses (Graham and Graham, 1996; Asai et al., 2000; Brodersen et al., 2002). qRT-PCR assays also showed that putative olive *CO-MT* and *GRAS1* genes were validated at all tested times indicating that, at least, the phenylpropanoid pathway and one TF related to defense response are systemically induced during a prolonged period of time upon *V. dahliae*-olive roots. On the contrary, validation of putative olive genes *ACL, ACO, DRR2, PR10*, and *DEF* was not achieved at all tested time points. This suggests that these genes are expressed in a transient way and/or at time points other than those chosen in this study.

The expression patterns of *ACO, ACL, GRAS1*, and *DRR2* in “Frantoio” plants in the second experiment showed some differences compared to that observed for this cultivar in the first experiment, particularly for *DRR2*. Plants used in these experiments showed the same appearance and were at the same phenological stage, although they slightly differed in age (3-month-old vs. 8-month-old). This difference should be irrelevant for a woody, long-living plant such as olive. So far, we do not have any clear answer to explain this outcome. However, commonalities were found depending on the VWO susceptibility/tolerance level of olive cultivars tested. For instance, we found differences between *GRAS1* and *DRR2* relative expression patterns along time in tolerant cultivars (“Frantoio” and “Changlot Real”) compared to that in susceptible (“Picual”) plants. Indeed, *GRAS1* showed a constant down-regulation, although displaying a slight trend to increase its expression from 2 to 10DAI in tolerant cultivars (a situation observed in the first experiment for “Frantoio” plants as well). In contrast, “Picual” plants showed a sharp shift form up-regulation (2DAI) to down-regulation (10DAI). The induction of a *GRAS1* homologous gene (*SLGRAS1*) was shown during the incompatible interaction of tomato (*Solanum lycopersicum* Mill) plants with *Pseudomonas syringe* pv. tomato (Mysore et al., 2002; Mayrose et al., 2006). Accumulation of *SLGRAS1* transcripts was also found during two incompatible interactions of resistant tomato plants with *Xanthomonas campestris* pv. vesicatoria (Mayrose et al., 2006). Accordingly, the expression pattern of this TF was different depending on the level of VWO susceptibility. We suggest that expression of olive *GRAS1* can be repressed along time after *V. dahliae* infection in susceptible plants, while in tolerant cultivars systemic expression of this gene will show a trend to be induced after pathogen infection. Likewise, the olive *DIR* gene *DRR2* was mostly observed to be up-regulated in “Picual” plants, in contrast to the overall down-regulation found in tolerant cultivars. This *DIR* gene was reported to be induced during the interaction of the resistant olive cultivar Moraiolo with *Bactrocera oleae* (olive fruit fly)**, although up-regulation of this gene was not validated (Corrado et al., 2012). Furthermore, some similarities can be established to the response of rice (*Oryza sativa* L.) to heat stress. Thus, Jagadish et al. (2010) reported that the amount of a DIR protein did not change significantly in an *O. sativa* heat-susceptible genotype, while in a moderately heat-tolerant genotype a significant decrease of this protein was observed. These findings seem to point to a down regulation of these genes to cope with different (a)biotic stresses.

No clear correlation was found between expression pattern of the rest of the evaluated genes and VWO susceptibility degree. Moreover, the two biological replicas (plants) tested per cultivar did not always show the same expression pattern. This could be due to differences in pathogen colonization (spatial and/or temporal) for each plant. However, absence of *V. dahliae* infection can be ruled out since pathogen DNA was detected in the roots of all sampled plants. Even though the complexity of the pathosystem under study (i.e., large and complex root systems, uneven vascular localization by the pathogen, non-gnotobiotic study system, etc.), consistent results were found in some cases. Thus, *ACO* expression pattern in “Frantoio” plants was similar in both experiments, with a trend to be down regulated from 2 to 10DAI. However, no clear correlation between its pattern and VWO tolerance was observed since “Picual” and “Changlot Real” plants showed the same expression trend for this gene along time. Accordingly, *ACO* is not a useful marker to differentiate between VWO susceptible and tolerant cultivars. Difference in *ACO* expression (and in *ACL* as well, see below) found between “Changlot Real” and “Frantoio” plants (Figure 3) indicate that level of tolerance to *V. dahliae* may not be the same for both cultivars, as otherwise observed for the progenies obtained from these cultivars when they are used as genitors (Trapero et al., 2015). Thus, while “Frantoio” produced a large number of VWO tolerant seedlings, even when crossed with the susceptible “Picual,” “Changlot Real” produced many *V. dahliae*-susceptible descendants.

A putative olive *ACL* gene was found in both “Frantoio” cDNA libraries. However, different accession numbers were found when searching databases (EYU44351 in FU library and XP_004234906 in FD library). Primer pair used in qRT-PCR experiments did not discriminate between them. A third ACL was found in FD library (FD05-F10T7) but its sequence was different. A possible explanation for the presence of putative olive *ACL* in both libraries is the existence of two different alleles/genes (74 SNPs in 597 nucleotides overlapped; data not shown). Each library was constructed from a pool of RNAs sampled along a 21-days interval after *V. dahliae* inoculation, and expression of this gene could undergo fluctuation (up and down regulation) along this time period. While *ACL* expression pattern showed a consistent trend in “Frantoio” plants in both experiments (a gene repression trend from 2 to 10DAI), results from the second experiment did not allow correlating olive cultivar susceptibility to *V. dahliae* with expression of this gene. Therefore, as for *ACO* expression, *ACL* does not help to differentiate between VWO susceptible and tolerant olive cultivars. Actually, expression of this gene showed, overall, erratic among cultivars tested and even between plants of the same cultivar. Induction of *ACL* gene has been previously reported at both local (Schilirò et al., 2012) and systemic (Gómez-Lama Cabanás et al., 2014) level in olive (cv. Arbequina) aerial tissues upon root colonization by the beneficial root endophytic bacterium *P. fluorescens* PICF7. Nevertheless, validation of *ACL* up-regulation was not possible in above-ground tissues (Gómez-Lama Cabanás et al., 2014), confirming the difficulty to unravel the role of this gene and/or supporting the actual presence of different *ACL* genes/alleles in olive. These findings encourage more in-depth studies on the actual role of this gene(s). Differential expression of *ACL* gene(s) in both beneficial (olive-endophytic bacterium) and pathogenic (olive-vascular fungal pathogen) interactions points out to the possibility that this gene may be involved in a general plant defense responses against colonization of different microorganisms.

In conclusion, we report for the first time the broad, systemic transcriptomic changes taking place during the interaction between a devastating vascular fungal pathogen and a VWO tolerant cultivar of a woody host of high commercial relevance (i.e., not a model plant). We have demonstrated that many of these changes are related to defense responses. From the genetic responses detected, the differential expression of *GRAS1* and *DRR2* genes observed in the olive cultivars here assessed deserves further attention to be explored as markers of the susceptibility/tolerance level of olive genotypes to VWO. Information here reported not only shed light on defense mechanisms operating in olive against *V. dahliae* attack, but also could pave the way to develop novel tools in breeding for VWO resistance and management of this vascular pathogen.

### Conflict of interest statement

The authors declare that the research was conducted in the absence of any commercial or financial relationships that could be construed as a potential conflict of interest.
