# Supplementary material for: Systemic responses in a tolerant olive (Olea europaea L.) cultivar upon root colonization by the vascular pathogen Verticillium dahliae
Source: Front Microbiol. 2015 Sep 16;6:928. doi: 10.3389/fmicb.2015.00928 (PMC4584997; doi:10.3389/fmicb.2015.00928)
Supplement: Supplementary file 1 [file Table1.DOC]

| **Table S1**. List of EST sequences induced in aerial olive tissues (cv. Frantoio) upon root inoculation with *Verticillium dahliae* defoliating pathotype. The EST Sequence name refers to the codes assigned within the cDNA library. FU means Frantoio aerial tissues induced gene and FU-C indicates Frantoio aerial tissues inducted as part of a contig. T7 refers to the forward T7 universal primers used for sequencing. Homologous genes were identified in the GenBank protein database (non-redundant) by running the Blastx algorithm set to 1.0 E-3 (in Blast2GO pro trial). ESTs found in both libraries and ESTs homologous to protein with unknown function are not included in this table. EST sequence name, putative protein function, organism, accession number, and related E-value are shown. ESTs in bold type letters were selected for validation and relative gene expression studies. | | | | |
| --- | --- | --- | --- | --- |
| **EST Sequence name** | **Putative protein function** | **Organism** | **Accession Number** | **E-Value** |
| FU01-A04T7 | dicarboxylate transporter chloroplastic-like | *Populus trichocarpa* | XP-006379073 | 2.46E-47 |
| FU01-A05T7 | hypothetical protein MIMGU-mgv1a005588mg | *Erythranthe guttata* | EYU20143 | 1.69E-09 |
| FU01-A07T7 | protein dehydration-induced 19 homolog 4-like | *Nicotiana sylvestris* | XP-009767629 | 4.70E-53 |
| FU01-A10T7 | chlorophyll a b binding protein | *Capsicum annuum* | AHI85722 | 3.60E-44 |
| FU01-A11T7 | hypothetical protein MIMGU-mgv1a026174mg | *Erythranthe guttata* | EYU23743 | 1.27E-40 |
| FU01-A12T7 | protein fam133-like | *Ricinus communis* | XP-002522053 | 2.30E-05 |
| FU01-B01T7 | sensory transduction histidine isoform 1 | *Coffea canephora* | CDP04335 | 2.54E-26 |
| FU01-B03T7 | vacuolar-processing enzyme-like | *Erythranthe guttata* | EYU20379 | 3.78E-42 |
| FU01-B09T7 | (-)-isopiperitenol (-)-carveol mitochondrial-like | *Nicotiana sylvestris* | XP-009773744 | 3.70E-29 |
| FU01-B10T7 | cannabidiolic acid synthase-like | *Vitis vinifera* | CBI22013 | 7.51E-20 |
| FU01-C04T7 | vin3-like protein 2-like | *Coffea canephora* | CDP02986 | 1.53E-19 |
| FU01-C05T7 | long chain acyl- synthetase 2-like | *gGenlisea aurea* | EPS68372 | 2.59E-90 |
| FU01-C07T7 | otu domain-containing protein ddb-g0284757-like | *Erythranthe guttata* | EYU42842 | 1.78E-55 |
| FU10-C08T7 | cyclin 4 | *Glycine max* | XP-006595782 | 2.40E-46 |
| FU01-C12T7 | elongation factor beta-1 | *Lotus japonicus* | AFK47903 | 3.19E-03 |
| FU01-D01T7 | erwinia induced protein 2 | *Solanum tuberosum* | NP-001275181 | 6.03E-21 |
| FU01-D03T7 | probable rhamnose biosynthetic enzyme 1 | *Vitis vinifera* | CAN79484 | 2.76E-44 |
| FU01-D04T7 | nucleobase-ascorbate transporter 6-like isoform x1 | *Plantago major* | CAH58638 | 2.22E-42 |
| FU01-D05T7 | protein transport protein sec61 subunit beta-like | *Cicer arietinum* | XP-004504298 | 6.26E-09 |
| FU01-D07T7 | diphosphomevalonate decarboxylase-like | *Catharanthus roseus* | ADR65113 | 6.43E-101 |
| FU01-D08T7 | la protein 1 | *Nicotiana sylvestris* | XP-009782667 | 8.96E-38 |
| FU01-D10T7 | cytochrome c oxidase-assembly factor mitochondrial | *Nicotiana sylvestris* | XP-009622991 | 2.82E-16 |
| FU01-D11T7 | atp sulfurylase chloroplastic-like | *Malus domestica* | XP-008347628 | 3.12E-58 |
| FU01-D12T7 | formamidase isoform x1 | *Coffea canephora* | CDP02587 | 1.00E-03 |
| FU01-E02T7 | probable ubiquitin-conjugating enzyme e2 25 | *Coffea canephora* | CDP05916 | 2.35E-39 |
| FU01-E06T7 | methionine s-methyltransferase | *Erythranthe guttata* | EYU35047 | 2.34E-19 |
| FU01-E11T7 | **scarecrow-like protein 13 GRAS** | *Nicotiana sylvestris* | XP-009792736 | 4.49E-77 |
| FU01-F01T7 | feronia-like protein | *Arabidopsis lyrata* | ACI23970 | 1.99E-81 |
| FU01-F02T7 | ap-3 complex subunit mu | *Prunus persica* | XP-007205263 | 5.36E-80 |
| FU01-F11T7 | quinone oxidoreductase-like protein chloroplastic-like | *Genlisea aurea* | EPS62233 | 4.18E-19 |
| FU01-F12T7 | senescence regulator | *Phaseolus vulgaris* | XP-007147731 | 3.30E-07 |
| FU01-G03T7 | dolichyl-diphosphooligosaccharide--protein glycosyltransferase subunit 1a | *Phoenix dactylifera* | XP-008786685 | 1.40E-17 |
| FU01-G08T7 | hypothetical protein PHAVU-003G221800g | *Phaseolus vulgaris* | XP-007155677 | 1.53E-03 |
| FU01-G09T7 | wall-associated receptor kinase-like 14 | *Solanum tuberosum* | XP-006338251 | 4.37E-87 |
| FU01-G12T7 | 60s ribosomal protein l27-like | *Erythranthe guttata* | EYU32722 | 1.38E-30 |
| FU01-H06T7 | calcium-binding protein kic | *Solanum tuberosum* | XP-006362130 | 7.66E-55 |
| FU01-H07T7 | histone h2a | *Nicotiana sylvestris* | XP-009766769 | 1.50E-31 |
| FU01-H08T7 | cysteine desulfurase chloroplastic | *Erythranthe guttata* | EYU40685 | 1.32E-47 |
| FU01-H11T7 | btb poz domain-containing protein at1g67900-like | *Coffea canephora* | CDP13229 | 2.77E-32 |
| FU01-H12T7 | probable carboxylesterase 8 | *Erythranthe guttata* | EYU32026 | 2.70E-37 |
| FU02-A01T7 | atp-dependent clp protease proteolytic subunit-related protein chloroplastic | *Erythranthe guttata* | EYU39061 | 5.38E-63 |
| FU02-A03T7 | spx domain-containing membrane protein at4g22990-like isoform x1 | *Vitis vinifera* | XP-002282540 | 2.74E-132 |
| FU02-A05T7 | beta-galactosidase | *Coffea canephora* | CDP21667 | 7.01E-73 |
| FU02-A08T7 | uncharacterized methyltransferase chloroplastic-like | *Solanum lycopersicum* | XP-004242719 | 6.96E-144 |
| FU02-A09T7 | atp-nad kinase family protein | *Zea mays* | AFW65485 | 4.96E-58 |
| FU02-A12T7 | probable 6-phosphogluconolactonase chloroplastic | *Nicotiana tomentosiformis* | XP-009589243 | 2.38E-85 |
| FU02-B01T7 | heat shock protein 70 kda | *Zea mays* | CAA55184 | 2.34E-38 |
| FU02-B03T7 | rnase h family isoform 2 | *Erythranthe guttata* | EYU29701 | 2.83E-47 |
| FU02-B04T7 | 5-methylthioadenosine s-adenosylhomocysteine deaminase | *Solanum tuberosum* | XP-006363777 | 1.33E-65 |
| FU02-B06T7 | guanylate-binding family protein isoform 1 | *Nicotiana sylvestris* | XP-009761100 | 2.21E-08 |
| FU02-B09T7 | f-box protein at2g27310-like | *Vitis vinifera* | CBI32366 | 1.98E-63 |
| FU02-B11T7 | 2-methyl-6-phytyl- -hydroquinone chloroplastic | *Eucalyptus grandis* | XP-010038690 | 3.15E-53 |
| FU02-B12T7 | protein disulfide isomerase-like 1-4 | *Vitis vinifera* | CBI16310 | 8.91E-96 |
| FU02-C05T7 | protein dehydration-induced 19 homolog 3-like | *Citrus clementina* | XP-006433494 | 2.56E-33 |
| FU02-C09T7 | ankyrin repeat-containing protein at3g12360-like | *Vitis vinifera* | XP-002276402 | 4.16E-31 |
| FU02-D01T7 | pterin-4-alpha-carbinolamine dehydratase | *Solanum tuberosum* | XP-006365591 | 1.72E-63 |
| FU02-D02T7 | rna-binding glycine-rich protein | *Erythranthe guttata* | EYU32708 | 4.94E-50 |
| FU02-D06T7 | far1-related sequence isoform 1 | *Erythranthe guttata* | EYU44823 | 8.53E-88 |
| FU02-D07T7 | beta-glucosidase 12-like | *Olea europaea* | AAL93619 | 1.89E-41 |
| FU02-D08T7 | dag chloroplastic-like | *Nicotiana tomentosiformis* | XP-009624175 | 3.36E-46 |
| FU02-D12T7 | secoisolariciresinol dehydrogenase-like | *Jatropha curcas* | KDP34634 | 6.66E-43 |
| FU02-E01T7 | mitochondrial import inner membrane translocase subunit tim16-like | *Nicotiana sylvestris* | XP-009775690 | 1.72E-28 |
| FU02-E03T7 | polyubiquitin | *Capsicum annuum* | AHI85710 | 5.56E-105 |
| FU02-E05T7 | nudix hydrolase 2-like isoform x1 | *Nicotiana tomentosiformis* | XP-009625160 | 4.46E-29 |
| FU02-E07T7 | ndh-dependent cyclic electron flow 1 isoform 2 | *Theobroma cacao* | XP-007024241 | 4.01E-28 |
| FU02-E09T7 | caffeoylshikimate esterase | *Nicotiana sylvestris* | XP-009777258 | 1.58E-09 |
| FU02-E10T7 | lipoxygenase family protein | *Olea europaea* | ACD43483 | 1.81E-85 |
| FU02-E11T7 | probable protein phosphatase 2c 58 | *Jatropha curcas* | KDP24098 | 3.22E-98 |
| FU02-F02T7 | endo- -beta-d-glucanase-like | *Nicotiana tomentosiformis* | XP-009621644 | 2.80E-107 |
| FU02-F03T7 | 60s ribosomal protein l34-like | *Genlisea aurea* | EPS67435 | 2.15E-21 |
| FU02-F07T7 | ribonucleotide reductase beta subunit | *Populus tremula* | ACE96771 | 6.65E-95 |
| FU02-F08T7 | calcium-dependent protein kinase sk5-like | *Populus trichocarpa* | XP-002325726 | 4.58E-07 |
| FU02-F09T7 | 7-dehydrocholesterol reductase | *Erythranthe guttata* | EYU21205 | 7.64E-92 |
| FU02-F12T7 | probable wrky transcription factor 20 isoform x2 | *Coffea canephora* | CDP13745 | 5.12E-78 |
| FU02-G08T7 | histone | *Arabidopsis thaliana* | NP-195713 | 8.48E-43 |
| FU02-G10T7 | hypothetical protein JCGZ-12526 | *Jatropha curcas* | KDP32065 | 4.00E-16 |
| FU02-G11T7 | sec1 family transport protein sly1-like | *Nicotiana tomentosiformis* | XP-009601662 | 1.33E-22 |
| FU02-G12T7 | mitogen-activated protein kinase homolog ntf3 | *Coffea canephora* | CDP03486 | 4.56E-44 |
| FU02-H01T7 | nadp-malic enzyme | *Flaveria trinervia* | AAB19243 | 2.51E-66 |
| FU02-H08T7 | probable disease resistance protein at4g33300-like | *Coffea canephora* | CDP07611 | 3.49E-38 |
| FU02-H10T7 | alanine--trna ligase | *Erythranthe guttata* | EYU19755 | 1.80E-41 |
| FU02-H11T7 | transmembrane 9 superfamily member 4-like partial | *Celosia trigyna* | AHI59279 | 1.43E-40 |
| FU02-H12T7 | auxin-induced protein 22d-like | *Nicotiana tabacum* | AAD32145 | 9.95E-13 |
| FU03-A01T7 | 40s ribosomal protein s8-like | *Populus trichocarpa* | XP-006373483 | 1.88E-31 |
| FU03-A04T7 | glutathione s-transferase zeta class-like | *Populus trichocarpa* | XP-006385118 | 1.69E-63 |
| FU03-A05T7 | mitochondrial import inner membrane translocase subunit tim23-1-like | *Erythranthe guttata* | EYU45872 | 4.56E-07 |
| FU03-A06T7 | knox transcription factor | *Picea sitchensis* | ADE76332 | 2.49E-11 |
| FU03-A08T7 | laccase-15-like isoform x2 | *Erythranthe guttata* | EYU24319 | 7.89E-92 |
| FU03-A11T7 | 1-aminocyclopropane-1-carboxylate oxidase | *Phelipanche ramosa* | BAF33502 | 1.07E-42 |
| FU03-B03T7 | dynamin-like protein 6 isoform 1 | *Citrus sinensis* | XP-006478328 | 5.44E-03 |
| FU03-B05T7 | ribosome biogenesis protein nsa2 homolog | *Coffea canephora* | CDO97758 | 9.12E-37 |
| FU03-B06T7 | protein lsd1 | *Pyrus x bretschneideri* | XP-009362158 | 2.31E-38 |
| FU03-B07T7 | gtpase-activating protein gyp7 | *Morus notabilis* | XP-010090894 | 3.07E-70 |
| FU03-B12T7 | ubiquinol-cytochrome-c reductase complex assembly factor 1-like | *Solanum tuberosum* | XP-006357921 | 2.53E-35 |
| FU03-C10T7 | thaumatin-like protein | *Olea europaea* | E3SU11 | 5.49E-28 |
| FU03-C11T7 | late embryogenesis abundant hydroxyproline-rich glycoprotein isoform 1 | *Malus domestica* | XP-008343410 | 7.11E-04 |
| FU03-C12T7 | ubiquitin-conjugating enzyme e2 36 | *Brassica napus* | CDY26882 | 2.36E-51 |
| FU03-C06T7 | upf0664 stress-induced protein | *Phoenix dactylifera* | XP-008810339 | 5.74E-51 |
| FU03-D01T7 | squamosa promoter-binding-like protein 1 | *Vitis vinifera* | XP-002273228 | 9.37E-32 |
| FU03-D03T7 | actin-1 | *Gossypium thurberi* | AEN70747 | 2.45E-72 |
| FU03-D05T7 | protochlorophyllide-dependent translocon component chloroplastic-like | *Ocimum basilicum* | AII16851 | 3.62E-77 |
| FU03-D09T7 | calmodulin binding | *Lavandula angustifolia* | AHI49901 | 1.57E-08 |
| FU03-D11T7 | chaperone -domain superfamily protein | *Citrus clementina* | XP-006453013 | 3.13E-52 |
| FU03-D12T7 | membrane steroid-binding protein 2-like | *Jatropha curcas* | KDP22298 | 1.22E-64 |
| FU03-E02T7 | psi reaction center subunit ii | *Vitis vinifera* | XP-002281825 | 1.02E-39 |
| FU03-F03T7 | bel1-like homeodomain protein 1-like | *Solanum lycopersicum* | XP-004228612 | 4.98E-03 |
| FU03-F05T7 | late embryogenesis abundant protein lea5-like | *Nicotiana tomentosiformis* | XP-009596682 | 2.01E-14 |
| FU03-F06T7 | c-terminal processing chloroplastic-like | *Erythranthe guttata* | EYU43784 | 4.35E-50 |
| FU03-F09T7 | protein casp-like | *Coffea canephora* | CDO98449 | 9.30E-31 |
| FU03-F12T7 | homeobox-leucine zipper protein hat5-like | *Coffea canephora* | CDP00280 | 4.72E-30 |
| FU03-G03T7 | chloroplastic mitochondrial-like | *Erythranthe guttata* | EYU43668 | 2.04E-17 |
| FU03-G04T7 | ferredoxin- chloroplastic-like | *Morus notabilis* | XP-010101150 | 1.06E-39 |
| FU03-G06T7 | aldo keto | *Salvia miltiorrhiza* | EF666999 | 4E-165 |
| FU03-G09T7 | psbp-like protein chloroplastic | *Nicotiana sylvestris* | XP-009804509 | 4.25E-11 |
| FU03-G11T7 | e3 ubiquitin-protein ligase at3g02290-like | *Nicotiana tomentosiformis* | XP-009610647 | 1.10E-16 |
| FU03-H07T7 | 60s ribosomal protein l7-3-like | *Coffea canephora* | CDP14387 | 3.12E-134 |
| FU03-H08T7 | 40s ribosomal protein s11-like | *Eutrema salsugineum* | XP-006404910 | 1.82E-28 |
| FU03-H02T7 | sec14 cytosolic factor-like | *Prunus mume* | XP-008220913 | 1.86E-81 |
| FU03-H03T7 | PREDICTED: uncharacterized protein LOC103427126 | *Malus domestica* | XP-008363420 | 3.34E-26 |
| FU03-H11T7 | hypothetical protein MIMGU-mgv1a017997mg. partial | *Erythranthe guttata* | EYU25724 | 3.53E-03 |
| FU03-H12T7 | 60s ribosomal protein l3 | *Erythranthe guttata* | EYU33272 | 7.97E-73 |
| FU04-A04T7 | gaga-binding transcriptional activator | *Erythranthe guttata* | EYU36766 | 1.94E-53 |
| FU04-A06T7 | 3-isopropylmalate dehydratase large subunit-like | *Theobroma cacao* | XP-007035281 | 6.72E-68 |
| FU04-A11T7 | dna damage-inducible protein 1-like | *Solanum tuberosum* | XP-006352544 | 3.18E-10 |
| FU04-B06T7 | cation calcium exchanger 4-like | *Erythranthe guttata* | EYU21313 | 8.06E-52 |
| FU04-B07T7 | stromal cell-derived factor 2-like | *Ricinus communis* | XP-002515438 | 7.69E-67 |
| FU04-B08T7 | f-box lrr-repeat protein 23 | *Coffea canephora* | CDP20447 | 1.69E-10 |
| FU04-C02T7 | elongation factor 1-delta | *Solanum tuberosum* | NP-001274914 | 8.29E-45 |
| FU04-C03T7 | uncharacterized aarf domain-containing protein kinase chloroplastic | *Malus domestica* | XP-008380073 | 3.53E-48 |
| FU04-C04T7 | gtp-binding nuclear protein ran-3 | *Dimocarpus longan* | AFD93408 | 3.42E-63 |
| FU04-D04T7 | 40s ribosomal protein s6-like | *Ricinus communis* | XP-002525831 | 2.75E-03 |
| FU04-D05T7 | alcohol dehydrogenase | *Olea europaea* | AEQ04839 | 2.49E-91 |
| FU04-D08T7 | adp-ribosylation factor 2-like | *Vitis vinifera* | CBI25984 | 5.11E-103 |
| FU04-D09T7 | coronatine-insensitive protein 1-like | *Nicotiana attenuata* | ABK27928 | 6.70E-05 |
| FU04-G10T7 | 24-methylenesterol c-methyltransferase 2 | *Olea europaea subsp. europaea* | AGR55393 | 1.81E-74 |
| FU04-E07T7 | temperature-induced lipocalin | *Solanum tuberosum* | XP-006350147 | 9.69E-11 |
| FU04-F03T7 | nuclear transcription factor y subunit a-1-like | *Antirrhinum majus* | CAM12542 | 9.28E-23 |
| FU04-F05T7 | peptide methionine sulfoxide reductase b5-like | *Vitis vinifera* | CBI26152 | 5.07E-39 |
| FU04-F08T7 | serine decarboxylase | *Hirudo medicinalis* | CCJ09762 | 3.69E-34 |
| FU04-F09T7 | hva22-like protein a-like | *Erythranthe guttata* | EYU24390 | 1.01E-93 |
| FU04-F10T7 | 29 kda ribonucleoprotein chloroplastic | *Coffea canephora* | CDP06746 | 1.17E-43 |
| FU04-G03T7 | 3-hydroxy-3-methylglutaryl reductase 2 | *Gentiana lutea* | BAE92731 | 4.38E-18 |
| FU04-G04T7 | cytochrome p450 71a8-like | *Coffea canephora* | CDP19360 | 9.50E-113 |
| FU04-G06T7 | cannabidiolic acid synthase-like | *Nicotiana tomentosiformis* | XP-009619964 | 5.07E-56 |
| FU04-G07T7 | magnesium transporter mrs2-1-like | *Populus trichocarpa* | XP-006368495 | 2.37E-41 |
| FU04-H07T7 | rna-binding protein 25 isoform x2 | *Nicotiana tomentosiformis* | XP-009628935 | 5.71E-41 |
| FU04-H09T7 | peptidyl-prolyl cis-trans isomerase cyp19-4-like | *Panax ginseng* | AIC33037 | 2.29E-21 |
| FU05-A01T7 | 1-aminocyclopropane-1-carboxylate oxidase | *Vitis vinifera* | XP-002273430 | 9.55E-41 |
| FU05-A02T7 | 6-phosphogluconate decarboxylating 3-like | *Ricinus communis* | XP-002530803 | 1.67E-81 |
| FU05-A06T7 | 2-oxoisovalerate dehydrogenase subunit beta mitochondrial-like | *Erythranthe guttata* | EYU39280 | 1.12E-93 |
| FU05-A07T7 | methylcrotonoyl- carboxylase beta mitochondrial-like | *Erythranthe guttata* | EYU37666 | 1.65E-108 |
| FU05-A08T7 | far-red elongated hypocotyls 3 isoform 3 | *Erythranthe guttata* | EYU42833 | 1.74E-108 |
| FU05-B02T7 | ubiquitin-conjugating enzyme e2 32-like | *Coffea canephora* | CDO98774 | 4.24E-12 |
| FU05-B03T7 | hydrogen-transporting atp rotational | *Populus trichocarpa* | XP-002324755 | 2.19E-59 |
| FU05-B04T7 | calcium binding protein | *Solanum tuberosum* | XP-006362329 | 1.17E-30 |
| FU05-B05T7 | mlo-like protein 1 | *Theobroma cacao* | XP-007029886 | 4.34E-59 |
| FU05-B08T7 | probable pectinesterase pectinesterase inhibitor 54-like | *Erythranthe guttata* | EYU28292 | 2.75E-19 |
| FU05-B09T7 | cyclin-dependent protein kinase inhibitor smr2-like | *Vitis vinifera* | XP-002273869 | 4.82E-11 |
| FU05-B11T7 | atp-dependent protease la domain-containing protein | *Citrus sinensis* | KDO84696 | 4.57E-18 |
| FU05-C02T7 | zinc finger protein constans-like 16-like | *Nicotiana tomentosiformis* | XP-009627333 | 5.66E-24 |
| FU05-C05T7 | splicing factor 3b subunit 5 rds3 complex subunit 10 | *Coffea canephora* | CDO99245 | 1.43E-34 |
| FU05-C06T7 | carbamoyl-phosphate synthase large chloroplastic-like | *Coffea canephora* | CDP18860 | 5.64E-83 |
| FU05-C12T7 | sucrose phosphate synthase | *Craterostigma plantagineum* | O04932 | 1.26E-51 |
| FU05-D05T7 | gdt1-like protein 4 | *Coffea canephora* | CDP00587 | 7.77E-49 |
| FU05-D06T7 | suppressor of lin-12-like family protein | *Nicotiana sylvestris* | XP-009780768 | 1.01E-57 |
| FU05-D08T7 | somatic embryogenesis receptor kinase 1-like | *Vitis vinifera* | XP-002283683 | 1.35E-43 |
| FU05-D12T7 | tubby like protein 3 isoform 1 | *Vitis vinifera* | XP-002269323 | 9.75E-44 |
| FU05-E01T7 | lipid transfer protein | *Olea europaea* | ABS72013 | 6.81E-19 |
| FU05-E03T7 | sec14 cytosolic factor family protein phosphoglyceride transfer family protein | *Erythranthe guttata* | EYU20559 | 1.67E-75 |
| FU05-E04T7 | 40s ribosomal protein s13-2 | *Brassica napus* | CDX74728 | 2.12E-39 |
| FU05-E08T7 | pollen-specific protein sf21-like | *Solanum lycopersicum* | XP-004239328 | 1.85E-15 |
| FU05-E12T7 | beta-d-xylosidase family protein | *Populus trichocarpa x Populus deltoides* | ABK96385 | 3.08E-71 |
| FU05-F01T7 | family protein | *Theobroma cacao* | XP-007037807 | 1.83E-33 |
| FU05-F02T7 | acyl-coenzyme a oxidase peroxisomal-like | *Erythranthe guttata* | EYU41916 | 9.02E-45 |
| FU05-F08T7 | receptor-like protein kinase hsl1 | *Erythranthe guttata* | EYU29298 | 8.26E-11 |
| FU05-G06T7 | gdsl esterase lipase at1g29670-like | *Nicotiana sylvestris* | XP-009769555 | 2.36E-61 |
| FU05-G07T7 | cellulose synthase-like protein e6 | *Ricinus communis* | XP-002522780 | 4.42E-55 |
| FU05-G08T7 | malate dehydrogenase | *Populus trichocarpa* | XP-002312583 | 5.54E-169 |
| FU05-H03T7 | tubulin-folding cofactor a-like | *Jatropha curcas* | KDP39604 | 2.21E-56 |
| FU05-H07T7 | bystin-like isoform x1 | *Coffea canephora* | CDP06105 | 7.95E-57 |
| FU05-H08T7 | cellulose synthase-like protein | *Nicotiana tabacum* | AAZ79231 | 8.42E-75 |
| FU05-H09T7 | zinc finger ccch domain-containing protein 43-like | *Erythranthe guttata* | EYU40118 | 1.47E-34 |
| FU05-H12T7 | cation transport regulator-like protein 2 | *Erythranthe guttata* | EYU22079 | 1.35E-24 |
| FU06-A03T7 | ethylene-responsive transcription factor rap2-12-like | *Nicotiana tomentosiformis* | XP-009604966 | 4.36E-43 |
| FU06-A05T7 | lysyl-trna synthetase 1 isoform 3 | *Erythranthe guttata* | EYU40781 | 2.84E-145 |
| FU06-A07T7 | swib mdm2 domain superfamily protein | *Erythranthe guttata* | EYU37600 | 1.13E-23 |
| FU06-A08T7 | ptb domain-containing engulfment adapter protein 1 isoform 1 | *Nicotiana tomentosiformis* | XP-009593949 | 1.46E-22 |
| FU06-A10T7 | histidinol-phosphate aminotransferase | *Prunus persica* | XP-007200270 | 6.58E-34 |
| FU06-A12T7 | zinc knuckle family protein isoform 2 | *Coffea canephora* | CDO96994 | 6.00E-57 |
| FU06-B04T7 | wrky transcription factor 44-like isoform x1 | *Coffea canephora* | CDP06696 | 5.36E-03 |
| FU06-B06T7 | 40s ribosomal protein s19-3-like | *Erythranthe guttata* | EYU44178 | 7.48E-14 |
| FU06-B11T7 | chloroplast chlorophyll a b binding protein | *Helianthus annuus* | ABX71549 | 7.39E-74 |
| FU06-B12T7 | duf868 family protein | *Nicotiana sylvestris* | XP-009776174 | 4.91E-19 |
| FU06-C02T7 | skp1-like protein 1a | *Musa acuminata subsp. malaccensis* | XP-009401439 | 3.56E-36 |
| FU06-C04T7 | beta-glucosidase 12-like | *Olea europaea* | AAL93619 | 1.49E-41 |
| FU06-C05T7 | serine--glyoxylate aminotransferase | *Calycanthus occidentalis* | AGL09524 | 1.64E-35 |
| FU06-C10T7 | 4-hydroxy-4-methyl-2-oxoglutarate aldolase 2 | *Gossypium hirsutum* | ACJ11725 | 2.13E-76 |
| FU06-C11T7 | 60s ribosomal protein l28-1-like | *Solanum lycopersicum* | XP-004235619 | 1.52E-81 |
| FU06-D03T7 | cell division protein homolog 2- chloroplastic-like | *Nicotiana tabacum* | XP-009802019 | 3.09E-139 |
| FU06-D08T7 | fructokinase 2 | *Nicotiana sylvestris* | XP-009787481 | 2.00E-03 |
| FU06-D12T7 | 60s ribosomal protein l27a-3-like | *Coffea canephora* | CDP13218 | 2.82E-52 |
| FU06-E09T7 | glucose-6-phosphate 1- cytoplasmic isoform | *Eucalyptus grandis* | KCW55885 | 1.21E-63 |
| FU06-F01T7 | elongation factor 1-alpha | *Cicer arietinum* | CAA09041 | 1.22E-42 |
| FU06-F03T7 | autophagy-related protein 8f-like | *Eucalyptus grandis* | XP-010056366 | 2.17E-52 |
| FU06-F04T7 | glucose-6-phosphate isomerase chloroplastic-like isoform x2 | *Malus domestica* | XP-008378564 | 3.66E-69 |
| FU06-F09T7 | cobra-like protein 7 | *Coffea canephora* | CDO98803 | 2.13E-76 |
| FU06-F12T7 | rer1 family protein isoform 2 | *Hordeum vulgare subsp. vulgare* | BAK04477 | 1.98E-07 |
| FU06-G02T7 | protein phloem protein 2-like a1-like | *Populus trichocarpa* | XP-006377124 | 3.01E-08 |
| FU06-G05T7 | dnaj protein homolog | *Nicotiana tomentosiformis* | XP-009588725 | 3.82E-62 |
| FU06-G06T7 | fruit protein pkiwi502 | *Coffea canephora* | CDP00717 | 1.02E-23 |
| FU06-G09T7 | protein dj-1 homolog d-like | *Coffea canephora* | CDP01708 | 1.38E-50 |
| FU06-H01T7 | 1-aminocyclopropane-1-carboxylate oxidase homolog 1-like | *Theobroma cacao* | XP-007035064 | 1.11E-49 |
| FU06-H02T7 | probable peptide nitrate transporter at5g62680-like | *Nicotiana tomentosiformis* | XP-009623435 | 3.31E-52 |
| FU06-H06T7 | polygalacturonase at1g48100-like | *Nicotiana tomentosiformis* | XP-009629756 | 4.11E-92 |
| FU07-A02T7 | tom1-like protein 2 | *Nicotiana tomentosiformis* | XP-009623811 | 2.50E-26 |
| FU07-A06T7 | unnamed protein product | *Coffea canephora* | CDO99140 | 6.13E-16 |
| FU07-A04T7 | rhodanese-like domain-containing protein chloroplastic | *Coffea canephora* | CDP08699 | 2.78E-21 |
| FU07-A08T7 | cold-inducible rna-binding protein b-like | *Coffea canephora* | CDP18212 | 9.50E-03 |
| FU07-A09T7 | nadp-dependent malic enzyme | *Erythranthe guttata* | EYU27276 | 3.24E-76 |
| FU07-A10T7 | plastid-targeted protein 2 | *Erythranthe guttata* | EYU29383 | 7.05E-31 |
| FU07-A11T7 | 40s ribosomal protein s29-like | *Eucalyptus grandis* | KCW68667 | 9.25E-26 |
| FU07-B07T7 | alpha- -glucan-protein synthase | *Erythranthe guttata* | EYU39764 | 3.52E-74 |
| FU07-B10T7 | low quality protein: midasin-like | *Vitis vinifera* | XP-002274489 | 8.15E-30 |
| FU07-B12T7 | translocon-associated protein subunit beta-like | *Nicotiana sylvestris* | XP-009771788 | 7.70E-83 |
| FU07-C01T7 | ubiquitin-associated domain-containing family protein | *Nicotiana sylvestris* | XP-009797325 | 2.02E-29 |
| FU07-C06T7 | lysosomal pro-x carboxypeptidase-like | *Erythranthe guttata* | EYU25337 | 1.37E-34 |
| FU07-C07T7 | geranylgeranyl diphosphate synthase | *Scoparia dulcis* | AGV34300 | 1.79E-64 |
| FU07-C09T7 | late blight resistance protein homolog r1a-3 | *Coffea canephora* | CDP05558 | 1.46E-03 |
| FU07-C11T7 | hypersensitive-induced response protein 1 | *Jatropha curcas* | KDP31207 | 1.41E-69 |
| FU07-D06T7 | transmembrane protein 18-like | *Erythranthe guttata* | EYU23944 | 1.19E-60 |
| FU07-D08T7 | inositol transporter 1 | *Ricinus communis* | XP-002529745 | 1.16E-59 |
| FU07-D09T7 | 26s proteasome regulatory subunit 4 homolog a | *Citrus sinensis* | KDO65849 | 7.04E-76 |
| FU07-D11T7 | 60s ribosomal protein l11-1-like | *Arabidopsis lyrata subsp. lyrata* | XP-002880003 | 3.15E-51 |
| FU07-D12T7 | ubiquitin receptor rad23d-like isoform x1 | *Coffea canephora* | CDP02781 | 4.44E-104 |
| FU07-E03T7 | stress enhanced protein chloroplastic-like isoform x1 | *Solanum lycopersicum* | XP-004230742 | 8.16E-33 |
| FU07-E04T7 | suppressor of gene silencing | *Coffea canephora* | CDO99880 | 3.20E-30 |
| FU07-E06T7 | ribosomal protein l14 | *Staphylococcus aureus subsp. aureus* | CDP57011 | 8.14E-18 |
| FU07-E07T7 | Uncharacterized protein TCM-036395 | *Theobroma cacao* | XP-007020021 | 5.82E-11 |
| FU07-F02T7 | guanylate kinase 2-like | *Nicotiana tabacum* | AAG12251 | 5.67E-54 |
| FU07-F05T7 | uncharacterized loc101209678 isoform 1 | *Fragaria vesca subsp. vesca* | XP-004300691 | 2.36E-27 |
| FU07-F06T7 | uncharacterized partial | *Fragaria vesca subsp. vesca* | XP-004300003 | 3.43E-14 |
| FU07-F07T7 | 26s proteasome non-atpase regulatory subunit 11 homolog | *Coffea canephora* | CDP00375 | 7.43E-115 |
| FU07-G01T7 | formin-binding protein 4 isoform x1 | *Nicotiana tomentosiformis* | XP-009594051 | 3.08E-03 |
| FU07-G03T7 | photosystem ii 32 kda partial | *Imbribryum alpinum* | AAN85795 | 7.08E-86 |
| FU07-G05T7 | f-box protein 7 | *Citrus sinensis* | KDO80619 | 9.79E-87 |
| FU07-H02T7 | auxin-responsive protein iaa14-like | *Vitis vinifera* | XP-002284133 | 3.92E-92 |
| FU07-H03T7 | macrophage migration inhibitory factor homolog | *Erythranthe guttata* | EYU41965 | 7.00E-46 |
| FU07-H05T7 | dna-directed rna polymerase iv subunit 1 | *Nicotiana tomentosiformis* | XP-009593317 | 2.90E-05 |
| FU07-H06T7 | probable inactive shikimate kinase like chloroplastic | *Solanum tuberosum* | XP-006352906 | 2.29E-78 |
| FU07-H07T7 | stem-specific protein tsjt1-like | *Ricinus communis* | XP-002519037 | 1.14E-13 |
| FU07-H10T7 | light-inducible protein cprf2-like | *Vitis vinifera* | CBI34161 | 6.73E-31 |
| FU07-H12T7 | nsp-interacting kinase 3 isoform 2 | *Theobroma cacao* | XP-007044709 | 1.22E-25 |
| FU08-A05T7 | nuclear pore membrane glycoprotein 210-like | *Malus domestica* | XP-008341707 | 1.61E-76 |
| FU08-A07T7 | calmodulin | *Citrus sinensis* | XP-006470429 | 7.00E-70 |
| FU08-A10T7 | probable esterase kai2 | *Theobroma cacao* | XP-007047454 | 7.32E-11 |
| FU08-B01T7 | polyadenylate-binding protein rbp47 isoform x1 | *Nicotiana tomentosiformis* | XP-009628458 | 7.10E-21 |
| FU08-B02T7 | peptidyl-prolyl cis-trans isomerase fkbp12 | *Vitis vinifera* | NP-001267979 | 2.59E-47 |
| FU08-B05T7 | calmodulin binding | *Vitis vinifera* | XP-002279925 | 3.35E-62 |
| FU08-B06T7 | alpha-soluble nsf attachment protein | *Nicotiana sylvestris* | XP-009798526 | 1.72E-08 |
| FU08-B07T7 | cinnamoyl- reductase 1-like | *Coffea canephora* | CDP09238 | 1.36E-16 |
| FU08-C01T7 | ethylene insensitive 3-like 1 protein | *Nicotiana tabacum* | ADZ97022 | 5.51E-58 |
| FU08-C02T7 | eukaryotic translation initiation factor 3 subunit d-like | *Nicotiana sylvestris* | XP-009758822 | 6.15E-31 |
| FU08-D02T7 | nad -linked oxidoreductase superfamily protein | *Coffea canephora* | CDP06796 | 1.28E-53 |
| FU08-D04T7 | serine mitochondrial-like | *Olea europaea* | ABS72016 | 7.01E-136 |
| FU08-D05T7 | **caffeoyl- o-methyltransferase** | *Broussonetia papyrifera* | AAT37172 | 2.70E-67 |
| FU08-E01T7 | hydroquinone glucosyltransferase-like | *Forsythia x intermedia* | BAI65909 | 4.87E-44 |
| FU08-E10T7 | metallothionein-like protein | *Ilex paraguariensis* | AFP93964 | 1.66E-12 |
| FU08-E12T7 | desiccation-related protein pcc13-62-like | *Nicotiana sylvestris* | XP-009780622 | 3.96E-18 |
| FU08-F03T7 | kinase superfamily protein isoform 1 | *Coffea canephora* | CDO98324 | 8.65E-110 |
| FU08-G04T7 | myb-like protein x-like isoform x1 | *Theobroma cacao* | XP-007041417 | 2.48E-05 |
| FU08-G08T7 | 26s proteasome regulatory non-atpase rpn2 psmd1 subunit | *Ricinus communis* | XP-002518109 | 1.55E-88 |
| FU08-G10T7 | ctc-interacting domain 11 isoform 3 | *Erythranthe guttata* | EYU19687 | 4.38E-23 |
| FU08-H04T7 | nodulin-like major facilitator superfamily protein | *Erythranthe guttata* | EYU24433 | 9.30E-24 |
| FU08-H07T7 | mitotic-spindle organizing protein 1a-like | *Coffea canephora* | CDP14679 | 4.91E-23 |
| FU08-H09T7 | lipoxygenase homology domain-containing protein 1-like | *Erythranthe guttata* | EYU20207 | 1.76E-62 |
| FU09-A04T7 | vacuolar protein sorting-associated protein 45 homolog | *Nicotiana tomentosiformis* | XP-009629289 | 2.14E-46 |
| FU09-A05T7 | proteasome subunit alpha type-7 | *Erythranthe guttata* | EYU37945 | 1.38E-52 |
| FU09-A06T7 | ubiquitin-conjugating enzyme e2 28-like | *Erythranthe guttata* | EYU40353 | 1.10E-79 |
| FU09-A08T7 | u6 snrna-associated sm-like protein lsm1 | *Glycine max* | NP-001237465 | 2.70E-51 |
| FU09-A09T7 | e3 ubiquitin-protein ligase upl1-like isoform x2 | *Citrus sinensis* | KDO81247 | 4.43E-57 |
| FU09-B02T7 | lish domain and heat repeat-containing protein kiaa1468 homolog | *Nicotiana sylvestris* | XP-009765047 | 1.65E-61 |
| FU09-B06T7 | mitochondrial import receptor subunit tom6 homolog | *Morus notabilis* | XP-010096240 | 2.06E-13 |
| FU09-C02T7 | isoform 1 | *Coffea canephora* | CDP09163 | 6.38E-133 |
| FU09-C04T7 | cbl-interacting protein kinase 3 | *Citrus sinensis* | KDO66335 | 4.84E-77 |
| FU09-C05T7 | proline--trna ligase | *Nicotiana tomentosiformis* | XP-009600189 | 2.77E-95 |
| FU09-D01T7 | mitochondrial-processing peptidase subunit alpha-like | *Citrus sinensis* | KDO55872 | 2.73E-41 |
| FU09-D07T7 | protein disulfide-isomerase-like | *Nicotiana tomentosiformis* | XP-009618352 | 1.12E-72 |
| FU09-D12T7 | fertilization-independent endosperm protein | *Erythranthe guttata* | EYU19208 | 2.60E-18 |
| FU09-E03T7 | palmitoyl-protein thioesterase 1-like | *Pyrus x bretschneideri* | XP-009334531 | 1.75E-03 |
| FU09-E06T7 | ferredoxin-thioredoxin variable chain-like | *Erythranthe guttata* | EYU29040 | 2.10E-21 |
| FU09-E04T7 | F17A17.10 protein | *Theobroma cacao* | XP-007049313 | 6.02E-68 |
| FU09-E10T7 | glutamine synthetase partial | *Arachis hypogaea* | ACF74298 | 6.34E-44 |
| FU09-F04T7 | phospholipid-transporting atpase 8 | *Ricinus communis* | XP-002524646 | 2.84E-10 |
| FU09-F10T7 | alpha- -glucan-protein synthase | *Malus domestica* | XP-008346784 | 1.36E-74 |
| FU09-H02T7 | polyamine oxidase 2 | *Citrus sinensis* | KDO44819 | 1.98E-17 |
| FU09-H03T7 | mediator-associated protein 1-like | *Prunus persica* | XP-007214850 | 2.34E+01 |
| FU09-H07T7 | ribosome production factor 2 homolog | *Nicotiana tomentosiformis* | XP-009601445 | 1.06E-17 |
| FU09-H11T7 | protein kinase chloroplastic-like | *Erythranthe guttata* | EYU18976 | 2.66E-44 |
| FU10-A04T7 | f-box protein at2g27310-like | *Erythranthe guttata* | EYU36048 | 2.47E-18 |
| FU10-C01T7 | pyruvate dehydrogenase e1 component subunit mitochondrial | *Eucalyptus grandis* | XP-010044670 | 3.59E-15 |
| FU10-C03T7 | proteasome inhibitor-related | *Nicotiana sylvestris* | XP-009770213 | 5.97E-20 |
| FU10-C05T7 | cysteine proteinase 15a-like | *Phaseolus vulgaris* | XP-007160703 | 3.89E-19 |
| FU10-D02T7 | membrane steroid-binding protein 2-like | *Erythranthe guttata* | EYU35661 | 1.19E-57 |
| FU10-D09T7 | stress response protein nst1-like | *Theobroma cacao* | XP-007024476 | 3.52E-59 |
| FU10-E03T7 | c2 domain-containing protein | *Nicotiana tomentosiformis* | XP-009602442 | 1.37E-11 |
| FU10-E06T7 | pleiotropic drug resistance protein 1-like | *Erythranthe guttata* | EYU29215 | 1.53E-27 |
| FU10-E09T7 | membrane bound nac transcription factor 2 | *Petunia x hybrida* | AAM34770 | 2.08E-17 |
| FU10-F05T7 | cbl-interacting serine threonine-protein kinase 1-like | *Jatropha curcas* | KDP28011 | 1.46E-111 |
| FU10-F08T7 | protein kinase chloroplastic-like | *Nicotiana tomentosiformis* | XP-009590721 | 2.98E-57 |
| FU10-F09T7 | 60s ribosomal protein l44 | *Solanum lycopersicum* | XP-004236215 | 3.81E-37 |
| FU10-F10T7 | transcription factor ice1-like | *Jatropha curcas* | KDP41846 | 2.61E-57 |
| FU10-H03T7 | e3 ubiquitin-protein ligase rha1b-like | *Nicotiana sylvestris* | XP-009800798 | 4.82E-33 |
| FU10-H06T7 | probable receptor-like protein kinase at1g67000-like | *Vitis vinifera* | XP-002267505 | 1.42E-29 |
| FU11-A09T7 | b chain structures of alkaloid biosynthetic glucosidases decode substrate specificity | *Olea europaea* | AAL93619 | 7.38E-44 |
| FU11-B01T7 | benzyl alcohol o-benzoyltransferase | *Vitis vinifera* | XP-002266114 | 2.83E-11 |
| FU11-B02T7 | beta-glucosidase 44-like | *Olea europaea* | AAL93619 | 1.19E-18 |
| FU11-B06T7 | exocyst complex component sec10 isoform 6 | *Coffea canephora* | CDP13409 | 1.52E-107 |
| FU11-B07T7 | cytochrome p450 cyp72a219-like isoform x2 | *Sesamum indicum* | AAZ07706 | 3.89E-66 |
| FU11-B11T7 | probable wrky transcription factor 33 | *Capsicum annuum* | ABD65255 | 3.35E-07 |
| FU11-C01T7 | s-adenosylmethionine decarboxylase proenzyme | *Theobroma cacao* | XP-007045669 | 3.64E-69 |
| FU11-C02T7 | wd40-like beta propeller repeat family protein | *Nicotiana tomentosiformis* | XP-009593179 | 8.32E-39 |
| FU11-C04T7 | probable steroid-binding protein 3-like | *Nicotiana sylvestris* | XP-009772850 | 4.84E-42 |
| FU11-D01T7 | cryptochrome-1 | *Nicotiana tomentosiformis* | XP-009623394 | 2.53E-03 |
| FU11-D06T7 | 60s ribosomal protein l19-2 | *Pinus lambertiana* | AEW08596 | 2.54E-23 |
| FU11-D08T7 | calcium-transporting atpase plasma membrane-type-like | *Populus trichocarpa* | XP-002322655 | 9.89E-22 |
| FU11-E05T7 | ubiquitin isoform 1 | *Coffea canephora* | CDO99257 | 6.09E-12 |
| FU11-E08T7 | hypothetical protein MIMGU-mgv1a026174mg | *Erythranthe guttata* | EYU23743 | 1.66E-26 |
| FU11-E10T7 | mitotic-spindle organizing protein 1a-like | *Coffea canephora* | CDP14679 | 3.96E-19 |
| FU11-F05T7 | calcium-transporting atpase endoplasmic reticulum-type-like | *Vitis vinifera* | CAN79679 | 1.92E-76 |
| FU11-F08T7 | chain a family protein | *Ricinus communis* | XP-002533800 | 4.05E-66 |
| FU11-F10T7 | ribulose- -bisphosphate carboxylase oxygenase activase | *Olea europaea* | ABS72022 | 5.60E-90 |
| FU11-F12T7 | dentin sialophospho | *Populus trichocarpa* | XP-002306159 | 2.80E-07 |
| FU11-H01T7 | protein pat1 homolog 1-like | *Solanum tuberosum* | XP-006351984 | 2.38E-24 |
| FU11-H07T7 | kh domain-containing protein at4g18375-like | *Vitis vinifera* | CBI30926 | 1.05E-20 |
| FU11-H09T7 | bsd domain-containing family protein | *Erythranthe guttata* | EYU44516 | 5.67E-50 |
| FU11-H10T7 | hypothetical protein MIMGU-mgv1a014963mg | *Erythranthe guttata* | EYU43174 | 9.04E-38 |
| FU11-H12T7 | probable 3-hydroxyisobutyrate dehydrogenase-like mitochondrial-like | *Erythranthe guttata* | EYU27269 | 1.40E-57 |
| FU12-A03T7 | cytochrome b5-like | *Olea europaea* | CAA04702 | 5.53E-14 |
| FU12-A11T7 | mads-box protein svp-like isoform x3 | *Solanum macrocarpon* | AAV65497 | 9.54E-63 |
| FU12-A12T7 | 4-hydroxy-3-methylbut-2-enyl diphosphate chloroplastic-like | *Mitragyna speciosa* | AFB70984 | 3.10E-23 |
| FU12-B01T7 | ankyrin repeat-containing protein 2 | *Erythranthe guttata* | EYU26543 | 4.19E-70 |
| FU12-B03T7 | zinc finger protein jackdaw-like | *Coffea canephora* | CDP06730 | 2.72E-04 |
| FU12-B05T7 | abc transporter c family member 10-like | *Vitis vinifera* | CBI22551 | 3.77E-26 |
| FU12-B08T7 | peptidyl-prolyl cis-trans isomerase fkbp65-like | *Jatropha curcas* | KDP45275 | 6.14E-03 |
| FU12-B11T7 | 1102209g orf 7 | *Nicotiana tabacum* | AAA84679 | 5.91E-08 |
| FU12-C02T7 | proteasome subunit alpha type-4 | *Populus trichocarpa* | XP-002307867 | 7.55E-127 |
| FU12-D05T7 | histone partial | *Medicago truncatula* | XP-003589385 | 2.77E-14 |
| FU12-D08T7 | acyl carrier protein mitochondrial | *Erythranthe guttata* | EYU40321 | 6.32E-46 |
| FU12-E08T7 | calmodulin-binding family protein | *Nicotiana tomentosiformis* | XP-009615272 | 2.77E-31 |
| FU12-E11T7 | small ubiquitin-related modifier 1 | *Phoenix dactylifera* | XP-008775626 | 3.25E-51 |
| FU12-F09T7 | beta-glucosidase isozyme 2 precursor | *Olea europaea* | AAL93619 | 8.19E-48 |
| FU12-F10T7 | catalase | *Prunus armeniaca* | AAN78323 | 4.29E-35 |
| FU12-G02T7 | probable galactinol--sucrose galactosyltransferase 6 | *Nicotiana tomentosiformis* | XP-009613354 | 2.35E-46 |
| FU12-G08T7 | cathepsin b-like | *Picrorhiza kurrooa* | AHL68670 | 1.71E-82 |
| FU12-H01T7 | 60s ribosomal protein l21-1-like | *Brassica napus* | CDY50892 | 1.64E-11 |
| FU12-H06T7 | golgin candidate 5 | *Nicotiana tomentosiformis* | XP-009624004 | 2.67E-97 |
| FU12-H11T7 | 60s ribosomal protein l35-like | *Phaseolus vulgaris* | XP-007142699 | 6.61E-10 |
| FU12-H12T7 | zinc finger ccch domain-containing protein 29-like | *Cucumis sativus* | XP-004163130 | 4.85E-28 |
| FU13-A02T7 | subtilisin-like protease-like | *Solanum tuberosum* | XP-006359680 | 2.75E-124 |
| FU13-A04T7 | probable glycerophosphoryl diester phosphodiesterase 3 | *Nicotiana tomentosiformis* | XP-009593560 | 1.05E-54 |
| FU13-A05T7 | er membrane protein complex subunit 4-like | *Coffea canephora* | CDP21110 | 1.54E-22 |
| FU13-A07T7 | receptor-like cytosolic serine threonine-protein kinase rbk2 isoform x1 | *Nicotiana tomentosiformis* | XP-009626408 | 1.93E-109 |
| FU13-A09T7 | cell division protein homolog chloroplastic-like | *Cicer arietinum* | XP-004500118 | 2.36E-63 |
| FU13-B05T7 | chlorophyll a b-binding protein cab- partial | *Citrus sinensis* | KDO78113 | 5.68E-29 |
| FU13-B07T7 | aspartate chloroplastic | *Nicotiana tabacum* | BAD02268 | 1.40E-51 |
| FU13-B10T7 | nad -binding rossmann-fold superfamily protein isoform 3 | *Phoenix dactylifera* | XP-008802556 | 1.95E-67 |
| FU13-C11T7 | atp synthase subunit mitochondrial-like | *Vitis vinifera* | XP-002284053 | 5.05E-16 |
| FU13-C02T7 | cytochrome p450 | *Hirudo medicinalis* | CCJ09771 | 2.73E-28 |
| FU13-D01T7 | 60s acidic ribosomal protein p0-like | *Nicotiana sylvestris* | XP-009791710 | 2.24E-61 |
| FU13-D06T7 | cytochrome p450 subunit cyp72a13 | *Olea europaea* | AFS28694 | 1.38E-21 |
| FU13-D08T7 | sodium calcium exchanger family protein calcium-binding ef hand family protein isoform 1 | *Solanum tuberosum* | XP-006348283 | 6.40E-08 |
| FU13-F03T7 | protein spiral1-like 3 | *Coffea canephora* | CDP18053 | 3.37E-27 |
| FU13-F04T7 | vesicle-associated membrane protein 726 | *Erythranthe guttata* | EYU46345 | 2.09E-40 |
| FU13-F10T7 | bark storage protein a-like | *Hirudo medicinalis* | CCJ09769 | 3.72E-77 |
| FU13-G01T7 | imidazoleglycerol-phosphate dehydratase | *Nicotiana tomentosiformis* | XP-009591437 | 1.52E-62 |
| FU13-G03T7 | btb poz domain-containing protein pob1-like | *Citrus sinensis* | KDO76413 | 1.17E-70 |
| FU13-G06T7 | chaperone protein dnaj chloroplastic-like | *Nicotiana sylvestris* | XP-009787630 | 1.65E-03 |
| FU13-G08T7 | ubiqp-horvu ame: full=polyubiquitin contains: ame: full=ubiquitin flags: partial | *Vernicia fordii* | AFJ04519 | 1.23E-91 |
| FU13-G10T7 | 26s proteasome non-atpase regulatory subunit 7 homolog a | *Erythranthe guttata* | EYU41265 | 1.24E-60 |
| FU13-H03T7 | d-galacturonate reductase-like | *Nicotiana sylvestris* | XP-009799045 | 1.46E-113 |
| FU13-H06T7 | b chain structures of alkaloid biosynthetic glucosidases decode substrate specificity | *Glicine max* | 4ATD-A | 3.49E-84 |
| FU13-H07T7 | hydroxycinnamoyl- shikimate quinate hydroxycinnamoyltransferase | *Coffea Canephora* | 4G0B-A | 9.58E-17 |
| FU14-A03T7 | axial regulator yabby 5 | *Glycine max* | ACU19358 | 7.86E-03 |
| FU14-A08T7 | 1-aminocyclopropane-1-carboxylate oxidase homolog 1-like | *Vitis vinifera* | CAN66006 | 8.03E-75 |
| FU14-B01T7 | hypersensitive-induced response protein 1-like | *Citrus sinensis* | KDO76049 | 1.75E-11 |
| FU14-B06T7 | trans- -dihydrobenzene- -diol dehydrogenase-like | *Solanum tuberosum* | XP-006355425 | 4.36E-21 |
| FU14-B10T7 | probable calcium-binding protein cml49 | *Phoenix dactylifera* | XP-008786888 | 1.03E-10 |
| FU14-C09T7 | oxidoreductase family protein | *Fragaria vesca subsp. vesca* | XP-004287257 | 1.38E-68 |
| FU14-D02T7 | polcalcin phl p 7-like | *Populus trichocarpa* | XP-006374952 | 1.65E-03 |
| FU14-D10T7 | vhs domain-containing protein at3g16270 | *Vitis vinifera* | XP-002284183 | 3.66E-45 |
| FU14-D11T7 | ribosomal protein l14 | *Boea hygrometrica* | YP-004940546 | 1.79E-20 |
| FU14-D12T7 | beta subunit isoform 1 | *Zea mays* | NP-001170748 | 2.24E-30 |
| FU14-F01T7 | pleiotropic drug resistance protein 1-like | *Erythranthe guttata* | EYU29215 | 2.83E-40 |
| FU14-F12T7 | heptahelical transmembrane protein2 | *Erythranthe guttata* | EYU34322 | 3.01E-60 |
| FU14-G04T7 | 40s ribosomal protein s3-3 | *Morus notabilis* | XP-010090123 | 1.54E-107 |
| FU14-G08T7 | phytochrome-associated serine threonine-protein phosphatase 3-like | *Nicotiana tomentosiformis* | XP-009612273 | 5.62E-50 |
| FU14-H01T7 | uncharacterized aarf domain-containing protein kinase 1-like | *Solanum tuberosum* | XP-006351500 | 1.30E-06 |
| FU14-H02T7 | carbonic chloroplast precursor | *Olea europaea* | CBL86547 | 2.77E-48 |
| FU-C1 | tyrosyl-dna phosphodiesterase 1-like | *Vitis vinifera* | CBI23170 | 1.01E-56 |
| FU-C10 | calmodulin | *Zea mays* | AFW78488 | 4.93E-72 |
| FU-C15 | guanylate-binding family protein isoform 1 | *Nicotiana sylvestris* | XP-009761100 | 5.19E-09 |
| FU-C17 | hypothetical protein. partial | *Olea europaea* | AFP49328 | 9.00E-13 |
| FU-C24 | pentatricopeptide repeat-containing protein chloroplastic-like | *Prunus persica* | XP-007205048 | 4.74E-124 |
| FU-C25 | transferring glycosyl groups | *Erythranthe guttata* | EYU28487 | 3.57E-82 |
| FU-C32 | cytochrome p450 94c1-like | *Nicotiana tomentosiformis* | XP-009589328 | 7.00E-17 |
| FU-C35 | glutaredoxin-c11-like | *Citrus sinensis* | XP-006444243 | 1.59E-56 |
| FU-C36 | chain a family protein | *Erythranthe guttata* | EYU44351 | 9.10E-12 |
| FU-C47 | sucrose-phosphatase 1 | *Erythranthe guttata* | EYU18148 | 5.92E-91 |
| FU-C50 | glutamate--glyoxylate aminotransferase 2 | *Cucumis melo* | XP-008467047 | 2.60E-108 |
| FU-C58 | hypothetical protein. partial | *Olea europaea* | AFP49328 | 1.01E-05 |
| FU-C59 | glutaredoxin family protein | *Coffea canephora* | CDP12180 | 8.72E-42 |
| FU-C64 | pentameric polyubiquitin | *Vernicia fordii* | AFJ04519 | 8.59E-97 |
| FU-C67 | tubby like protein 10 isoform 1 | *Citrus clementina* | XP-006432266 | 2.60E-72 |
| FU-C68 | early nodulin-like protein 2-like | *Vitis vinifera* | XP-002271669 | 1.87E-14 |
| FU-C72 | hypothetical protein LOTGIDRAFT-117410. partial | *Lottia gigantea* | XP-009054256 | 3.29E-03 |
| FU-C75 | poly -binding protein 4-like | *Erythranthe guttata* | EYU31912 | 2.85E-74 |
| FU-C92 | chloroplast acyl-acylcarrier protein thioesterase b | *Erythranthe guttata* | EYU23034 | 1.03E-93 |
| FU-C104 | high mobility group b protein 14 | *Nicotiana sylvestris* | XP-009789088 | 1.03E-40 |
| FU-C108 | palmitoyl-protein thioesterase 1-like | *Vitis vinifera* | CAN68811 | 1.72E-46 |
| FU-C111 | subunit b of the trimeric enzyme atp citrate lyase family protein | *Arabidopsis thaliana* | BAD93838 | 8.11E-36 |
| FU-C119 | nac domain-containing protein 19-like isoform x2 | *Nicotiana tomentosiformis* | XP-009613291 | 9.62E-17 |
| FU-C126 | root border cell-specific protein | *Erythranthe guttata* | EYU20669 | 2.31E-53 |
| FU-C129 | cor414-like partial | *Erythranthe guttata* | EYU28169 | 1.69E-24 |
| FU-C130 | phospholipase a1-iidelta-like | *Erythranthe guttata* | EYU41122 | 3.05E-46 |
| FU-C133 | ferredoxin-like protein | *Vitis vinifera* | XP-002281459 | 6.96E-74 |
| FU-C138 | probable nucleolar protein 5-2 | *Erythranthe guttata* | EYU23804 | 3.05E-81 |
| FU-C144 | chloroplast beta-amylase isoform 1 | *Theobroma cacao* | XP-007039632 | 2.94E-157 |
| FU-C145 | **salicylic acid-binding protein 2-like** | *Erythranthe guttata* | EYU44351 | 1.96E-23 |
| FU-C146 | metallothionein-like protein | *Plantago major* | CAH59436 | 1.05E-07 |
| FU-C147 | photosystem ii 22 kda protein | *Phoenix dactylifera* | XP-008783427 | 2.88E-74 |
| FU-C150 | hypothetical protein MIMGU-mgv1a012104mg | *Erythranthe guttata* | EYU39087 | 5.67E-50 |
| FU-C165 | pectin methylesterase | *Nicotiana benthamiana* | AAO85706 | 1.84E-15 |
| FU-C171 | 40s ribosomal protein s3a-like | *Coffea canephora* | CDO99096 | 2.01E-134 |
| FU-C191 | 60s ribosomal protein l11-1-like | *Theobroma cacao* | XP-007025337 | 8.15E-94 |
| FU-C195 | tho complex subunit 7 homolog | *Erythranthe guttata* | EYU46154 | 8.73E-113 |
| FU-C202 | 60s ribosomal protein l44 | *Coffea canephora* | CDP14595 | 1.21E-39 |
| FU-C203 | dna-directed rna polymerase i subunit rpa12-like | *Coffea canephora* | CDP07375 | 1.96E-46 |
| FU-C224 | linoleate 13s-lipoxygenase 2- chloroplastic-like | *Olea europaea* | ACD43485 | 3.09E-116 |
| FU-C228 | photosystem i subunit o-like | *Nicotiana tomentosiformis* | XP-009587811 | 1.25E-62 |
| FU-C235 | magnesium-protoporphyrin ix monomethyl ester | *Cucumis sativus* | XP-004144646 | 1.79E-50 |
| FU-C243 | splicing suppressor of white-apricot homolog isoform x2 | *Coffea canephora* | CDP13811 | 4.58E-20 |
| FU-C256 | peptidyl-prolyl cis-trans isomerase fkbp62-like | *Erythranthe guttata* | EYU39186 | 5.23E-04 |
| FU-C258 | alpha beta hydrolase fold superfamily | *Catharanthus roseus* | AAU95203 | 1.30E-80 |
| FU-C259 | phosphate transporter | *Solanum tuberosum* | XP-006354490 | 1.15E-06 |
