# Supplementary material for: Systemic responses in a tolerant olive (Olea europaea L.) cultivar upon root colonization by the vascular pathogen Verticillium dahliae
Source: Front Microbiol. 2015 Sep 16;6:928. doi: 10.3389/fmicb.2015.00928 (PMC4584997; doi:10.3389/fmicb.2015.00928)
Supplement: Supplementary file 2 [file Table2.DOC]

| **Table S2**. List of EST sequences repressed in aerial olive tissues (cv. Frantoio) upon root inoculation with *Verticillium dahliae* defoliating pathotype. The EST Sequence name refers to the codes assigned within the cDNA library. FD means Frantoio aerial tissues induced gene and FD-C indicates Frantoio aerial tissues repressed as part of a contig. T7 refers to the forward T7 universal primers used for sequencing. Homologous genes were identified in the GenBank protein database (non-redundant) by running the Blastx algorithm set to 1.0 E-3 (in Blast2GO pro trial). ESTs found in both libraries and ESTs homologous to protein with unknown function are not included in this table. EST sequence name, putative protein function, organism, accession number and related E-value are shown. ESTs in bold type letters were selected for validation and relative gene expression studies. | | | | |
| --- | --- | --- | --- | --- |
| **EST Sequence name** | **Putative protein function** | **Organism** | **Accession Number** | **E-Value** |
| FD01-A01T7 | aspartic proteinase-like protein 2-like | *Erythranthe guttata* | EYU39713 | 3.70E-04 |
| FD01-A04T7 | ubiquitin-conjugating enzyme e2-17 kda-like | *Jatropha curcas* | KDP21850 | 4.79E-60 |
| FD01-A05T7 | probable inactive purple acid phosphatase 27 | *Citrus cinensis* | KDO52666 | 1.17E-79 |
| FD01-A07T7 | hydroxypyruvate reductase | *Medicago truncatula* | ACJ85450 | 1.85E-29 |
| FD01-A09T7 | phosphatidylglycerol phosphatidylinositol transfer protein ddb_g0282179-like | *Nicotiana sylvestris* | XP_009789276 | 1.77E-18 |
| FD01-B03T7 | protein dehydration-induced 19 homolog 4-like | *Vitis vinifera* | CBI36908 | 1.55E-06 |
| FD01-B04T7 | nadp-dependent glyceraldehyde-3-phosphate dehydrogenase | *Platanus x acerifolia* | CAQ16337 | 4.31E-85 |
| FD01-C01T7 | alanine aminotransferase 2 | *Genlisea aurea* | EPS60556 | 4.56E-100 |
| FD01-C04T7 | vesicle-associated membrane protein 726 isoform 2 | *Erythranthe guttata* | EYU43691 | 1.81E-55 |
| FD01-C12T7 | alpha-l-arabinofuranosidase 1-like | *Jatropha curcas* | KDP35692 | 3.62E-09 |
| FD01-D03T7 | chlorophyll a-b binding protein chloroplastic-like | *Nicotiana sylvestris* | XP_009786355 | 4.94E-14 |
| FD01-D04T7 | e3 ubiquitin-protein ligase xbat31 | *Erythranthe guttata* | EYU36266 | 2.90E-32 |
| FD01-D05T7 | nadp-dependent d-sorbitol-6-phosphate dehydrogenase | *Nicotiana sylvestris* | XP_009804626 | 2.92E-30 |
| FD01-D06T7 | f-box protein pp2-b11-like | *Nicotiana tabacum* | AAZ81591 | 1.76E-16 |
| FD01-E01T7 | macrophage migration inhibitory factor homolog isoform x1 | *Jatropha curcas* | KDP22824 | 3.82E-47 |
| FD01-E04T7 | defective in exine formation protein isoform 1 | *Nicotiana tomentosiformis* | XP_009627437 | 1.88E-120 |
| FD01-E10T7 | bi1-like protein | *Arabidopsis thaliana* | NP_567466 | 1.38E-21 |
| FD01-E11T7 | protein chloroplastic | *Erythranthe guttata* | AFP49328 | 1.69E-42 |
| FD01-E12T7 | metallothionein-like protein | *Corchorus olitorius* | ABS72197 | 1.28E-12 |
| FD01-F06T7 | protein chloroplastic-like | *Olea europaea* | EYU17894 | 9.28E-43 |
| FD01-F09T7 | photosystem ii 10 kda chloroplastic | *Gossypium hirsutum* | AHK23268 | 2.10E-38 |
| FD01-F11T7 | early nodulin-like protein 2-like | *Theobroma cacao* | XP_007026253 | 4.14E-20 |
| FD01-G02T7 | peroxisome biogenesis protein 6-like | *Glycine max* | XP_006604705 | 7.86E-39 |
| FD01-G03T7 | chlorophyll a-b binding protein chloroplastic | *Vitis vinifera* | XP_002264295 | 1.49E-55 |
| FD01-G09T7 | ribulose bisphosphate carboxylase oxygenase activase chloroplast | *Erythranthe guttata* | EYU19932 | 7.67E-33 |
| FD01-H03T7 | photosystem i reaction center subunit chloroplastic-like | *Coffea canephora* | CDP17606 | 1.86E-63 |
| FD01-H06T7 | oxygen-evolving enhancer protein chloroplastic-like | *Lotus japonicus* | AFK44592 | 8.15E-89 |
| FD01-H08T7 | ole e 5 olive pollen allergen | *Olea europaea* | ABX26138 | 1.13E-45 |
| FD01-H10T7 | cbs domain-containing protein chloroplastic-like | *Genlisea aurea* | EPS68026 | 9.22E-50 |
| FD01-H12T7 | probable aquaporin tip1-1 | *Vitis vinifera* | CBI30163 | 2.55E-32 |
| FD02-A01T7 | inorganic phosphate transporter 1-4-like | *Solanum tuberosum* | XP_006354490 | 1.74E-46 |
| FD02-A07T7 | pre-mrna-splicing factor slu7-a-like | *Pyrus x bretschneideri* | XP_009369485 | 1.38E-04 |
| FD02-A06T7 | pectinesterase pectinesterase inhibitor u1 | *Erythranthe guttata* | EYU29495 | 1.06E-29 |
| FD02-A10T7 | chlorophyll a-b binding protein chloroplastic-like | *Musa acuminata subsp. malaccensis* | XP_009389379 | 2.36E-144 |
| FD02-A12T7 | unknown | *Populus trichocarpa* | ACU20555 | 9.94E-15 |
| FD02-B05T7 | 60s ribosomal protein l6-like | *Erythranthe guttata* | EYU22781 | 3.81E-63 |
| FD02-B10T7 | rna rnp complex-1-interacting | *Nicotiana sylvestris* | XP_009801567 | 4.58E-15 |
| FD02-C02T7 | glycerate dehydrogenase | *Fragaria vesca subsp. vesca* | XP_004287066 | 2.44E-47 |
| FD02-C03T7 | probable carboxylesterase 7-like | *Nicotiana sylvestris* | XP_009773904 | 7.40E-90 |
| FD02-C08T7 | probable cinnamyl alcohol dehydrogenase 1 | *Nicotiana tomentosiformis* | XP_009589196 | 2.19E-31 |
| FD02-C09T7 | serine carboxypeptidase-like | *Erythranthe guttata* | EYU37039 | 2.50E-64 |
| FD02-C11T7 | chaperone protein dnaj chloroplastic-like | *Erythranthe guttata* | EYU24590 | 1.26E-28 |
| FD02-D03T7 | cellulose synthase-like protein g3-like | *Nicotiana sylvestris* | XP_009793889 | 5.58E-05 |
| FD02-D04T7 | photosystem ii oxygen-evolving complex protein 2 precursor | *Morus notabilis* | XP_010093189 | 9.19E-26 |
| FD02-D05T7 | profilin | *Fragaria vesca subsp. vesca* | XP_004291152 | 2.33E-17 |
| FD02-D06T7 | apyrase 2-like | *Nicotiana tomentosiformis* | XP_009615583 | 2.05E-56 |
| FD02-D10T7 | uncharacterized loc101211262 | *Vitis vinifera* | CBI28559 | 3.70E-24 |
| FD02-E02T7 | chloroplast ribulose--bisphosphate carboxylase oxygenase small subunit | *Olea europaea* | ABS71998 | 3.00E-08 |
| FD02-E10T7 | myelin-associated oligodendrocyte basic protein isoform 2 | *Vitis vinifera* | CAN72331 | 6.44E-23 |
| FD02-F01T7 | glutathione s-transferase-like | *Prunus persica* | XP_007200490 | 2.07E-29 |
| FD02-F03T7 | membrane family protein | *Vitis vinifera* | CAN70428 | 4.33E-17 |
| FD02-G01T7 | protochlorophyllide reductase precursor-like protein | *Ricinus communis* | XP_002534245 | 2.59E-23 |
| FD02-G07T7 | AF197742_1maturase. partial (mitochondrion) | *Austrobaileya scandens* | AAF14734 | **2.17E-03** |
| FD02-G09T7 | carbonic anhydrase 2-like isoform x1 | *Striga asiatica* | ABE66400 | 9.63E-10 |
| FD02-H02T7 | homeobox-leucine zipper protein hat5-like | *Coffea canephora* | CDP00280 | 1.93E-09 |
| FD02-H03T7 | 5-formyltetrahydrofolate cyclo-ligase-like | *Nicotiana sylvestris* | XP_009768821 | 2.34E-10 |
| FD03-A06T7 | serine protease inhibitor family protein | *Theobroma cacao* | XP_007017373 | 7.66E-90 |
| FD03-A07T7 | glycine dehydrogenase | *Nicotiana benthamiana* | ADM18296 | 2.20E-66 |
| FD02-A08T7 | oxygen-evolving enhancer protein chloroplastic-like | *Nicotiana sylvestris* | XP_009759927 | 4.75E-84 |
| FD03-A10T7 | probable xyloglucan endotransglucosylase hydrolase protein 6 | *Pyrus x bretschneideri* | XP_009354593 | 3.84E-43 |
| FD03-B09T7 | probable lrr receptor-like serine threonine-protein kinase at4g36180-like | *Vitis vinifera* | XP_003634116 | 3.97E-17 |
| FD03-B10T7 | gdsl esterase lipase at2g04570-like | *Erythranthe guttata* | EYU26075 | 2.16E-62 |
| FD03-C06T7 | glycolate oxidase | *Olea europaea* | ABS72011 | 1.40E-67 |
| FD03-C07T7 | probable 6-phosphogluconolactonase chloroplastic | *Solanum lycopersicum* | XP_004240972 | 3.13E-19 |
| FD03-D04T7 | cytochrome b6-f complex subunit 7 | *Vitis vinifera* | XP_002284808 | 2.23E-10 |
| FD03-D10T7 | 20s proteasome beta subunit pbb2 | *Brassica napus* | CDY03969 | 2.00E-08 |
| FD03-D11T7 | zinc finger protein 622-like | *Vitis vinifera* | CBI20772 | 1.62E-73 |
| FD03-E06T7 | photosystem i reaction center subunit chloroplastic-like | *Olea europaea* | ABU39903 | 3.59E-48 |
| FD03-E09T7 | 40s ribosomal protein s6-like | *Citrus sinensis* | KDO48241 | 4.01E-05 |
| FD03-F06T7 | probable 26s proteasome complex subunit sem1-1 | *Nicotiana tomentosiformis* | XP_009600825 | 1.58E-10 |
| FD03-F07T7 | fatty acid hydroperoxide lyase | *Olea europaea* | ACD43482 | 6.90E-82 |
| FD03-F09T7 | 4-hydroxy-3-methylbut-2-enyl diphosphate partial | *Olea europaea* | AFS28680 | 3.21E-57 |
| FD03-G08T7 | senescence-associated family protein | *Rehmannia glutinosa* | AGC24177 | 1.05E-21 |
| FD03-G11T7 | 60s ribosomal protein l35-like | *Camellia sinensis* | AEC10955 | 3.62E-18 |
| FD03-H05T7 | s-adenosylmethionine-dependent methyltransferase | *Coffea canephora* | CDP04663 | 5.77E-07 |
| FD03-H11T7 | thylakoid lumenal kda chloroplastic-like | *Citrus clementina* | XP_006441152 | 5.89E-92 |
| FD03-H12T7 | dnaj protein homolog | *Coffea canephora* | CDP15561 | 8.07E-30 |
| FD04-B04T7 | protein yls9-like | *Fragaria vesca subsp. vesca* | XP_004303498 | 1.50E-30 |
| FD04-B08T7 | succinyl- ligase | *Morus notabilis* | XP_010087140 | 1.36E-09 |
| FD04-C07T7 | isopentenyl diphosphate isomerase | *Olea europaea* | AFS28681 | 1.99E-68 |
| FD04-C11T7 | eukaryotic translation initiation factor 5a-2 | *Nicotiana sylvestris* | XP_009758744 | 8.95E-36 |
| FD04-E04T7 | acid phosphatase 1-like | *Coffea canephora* | CDO97963 | 3.67E-24 |
| FD04-E10T7 | probable adp-ribosylation factor gtpase-activating protein agd11 | *Erythranthe guttata* | EYU23809 | 2.92E-39 |
| FD04-F04T7 | peptide-n4-(n-acetyl-beta-glucosaminyl)asparagine amidase a-like | *Erythranthe guttata* | EYU20053 | 7.90E-17 |
| FD04-F08T7 | cysteine proteinase | *Citrus sinensis* | KDO73909 | 4.03E-50 |
| FD04-F11T7 | somatic embryogenesis receptor-like kinase-like protein | *Helianthus annuus* | AAL93161 | 5.82E-80 |
| FD04-F12T7 | probable 6-phosphogluconolactonase chloroplastic | *Nicotiana sylvestris* | XP_009789475 | 6.02E-47 |
| FD04-H01T7 | **defensin ec-amp-d2-like** | *Olea europaea* | **ABS72000** | **1.66E-03** |
| FD04-H04T7 | protein yls9-like | *Coffea canephora* | CDP10610 | 6.89E-25 |
| FD04-H09T7 | raffinose synthase family protein | *Nicotiana tomentosiformis* | XP_009611189 | 6.47E-83 |
| FD05-A01T7 | small heat-shock | *Vitis vinifera* | XP_002282438 | 3.35E-68 |
| FD05-A04T7 | elongation factor 1- | *Ricinus communis* | XP_002538495 | 2.25E-64 |
| FD05-A10T7 | zinc finger ccch domain-containing protein 64 isoform x1 | *Erythranthe guttata* | EYU28403 | 2.02E-23 |
| FD05-B03T7 | dna binding | *Vitis vinifera* | CBI36325 | 1.46E-07 |
| FD05-B07T7 | protein exordium-like 3 | *Erythranthe guttata* | EYU43528 | 7.90E-53 |
| FD05-C09T7 | probable 6-phosphogluconolactonase chloroplastic-like | *Nicotiana sylvestris* | XP_009789475 | 8.45E-74 |
| FD05-D05T7 | aquaporin tip1-3-like | *Olea europaea* | ABB76813 | 2.30E-83 |
| FD05-F01T7 | n-carbamoylputrescine amidase-like | *Coffea canephora* | CDP06899 | 3.31E-42 |
| FD05-F06T7 | reticuline oxidase | *Erythranthe guttata* | EYU19096 | 6.97E-109 |
| FD05-F10T7 | salicylic acid-binding protein 2-like | *Coffea canephora* | CDP02455 | 7.70E-24 |
| FD05-G02T7 | atp synthase delta chloroplastic-like | *Coffea canephora* | CDP01168 | 1.90E-03 |
| FD05-G09T7 | mitochondrial import receptor subunit tom7-1-like | *Erythranthe guttata* | EYU26552 | 3.03E-21 |
| FD05-H09T7 | thiol protease aleurain-like | *Eucalyptus grandis* | XP_010050441 | 8.99E-13 |
| FD06-A02T7 | thioredoxin-dependent peroxidase | *Erythranthe guttata* | EYU32944 | 2.19E-98 |
| FD06-A06T7 | ref srpp-like protein at3g05500 | *Coffea canephora* | CDP18044 | 3.32E-05 |
| FD06-A07T7 | protein translation factor sui1 homolog | *Phaseolus vulgaris* | XP_007132952 | 2.58E-14 |
| FD06-B05T7 | thiosulfate 3-mercaptopyruvate sulfurtransferase mitochondrial-like | *Cicer arietinum* | XP_004488787 | 2.61E-38 |
| FD06-C03T7 | presequence protease chloroplastic mitochondrial | *Erythranthe guttata* | EYU37693 | **4.44E-03** |
| FD06-C09T7 | catalase | *Tectona grandis* | CBA13361 | 2.01E-55 |
| FD06-B12T7 | bzip transcription factor bzip133 | *Nicotiana tomentosiformis* | XP_009589375 | 1.56E-50 |
| FD06-D01T7 | profilin 1 isoform 1 | *Musa acuminata subsp. malaccensis* | XP_009405712 | 7.31E-17 |
| FD06-D06T7 | oxoglutarate dehydrogenase | *Eucalyptus grandis* | XP_010065984 | 7.43E-49 |
| FD06-D10T7 | ubiquitin carboxyl-terminal hydrolase 6 | *Erythranthe guttata* | EYU21397 | 5.61E-70 |
| FD06-E02T7 | ferredoxin-nadp reductase | *Coffea canephora* | CDP01892 | 1.11E-136 |
| FD06-E07T7 | cytochrome p450 isoform 2 | *Coffea canephora* | CDO97118 | 3.60E-10 |
| FD06-E08T7 | ubiquitin-conjugating enzyme e2-17 kda-like | *Jatropha curcas* | KDP21850 | 3.57E-61 |
| FD06-E12T7 | isopentenyl-diphosphate delta-isomerase i | *Olea europaea* | ACF05532 | 4.17E-10 |
| FD06-F07T7 | protein walls are thin 1-like | *Erythranthe guttata* | EYU32879 | 8.16E-15 |
| FD06-F12T7 | chloroplast chlorophyll a b binding protein | *Helianthus annuus* | ABX71549 | 1.33E-75 |
| FD06-G05T7 | photosystem i reaction center subunit chloroplastic-like | *Erythranthe guttata* | EYU27509 | 3.04E-55 |
| FD07-A06T7 | lipid transfer protein precursor | *Hirudo medicinalis* | CCJ09772 | 4.07E-33 |
| FD07-B04T7 | **1-aminocyclopropane-1-carboxylate oxidase** | *Plantago major* | **CAH58646** | **1.40E-49** |
| FD07-B09T7 | cytochrome p450 family protein | *Olea europaea* | AFS28690 | 8.18E-66 |
| FD07-B12T7 | peroxisomal membrane protein 11d-like | *Nicotiana tomentosiformis* | XP_009598453 | 5.19E-58 |
| FD07-C08T7 | photosystem i reaction center subunit chloroplastic-like | *Nicotiana tomentosiformis* | XP_009588187 | 5.77E-30 |
| FD07-C11T7 | polyubiquitin | *Medicago sativa* | AAZ32851 | 5.58E-103 |
| FD07-D02T7 | photosystem ii 23 kda polypeptide | *Lotus japonicus* | AFK44592 | 5.22E-45 |
| FD07-D07T7 | oxygen-evolving enhancer protein 3- chloroplastic-like | *Eucalyptus grandis* | XP_010052458 | 2.06E-26 |
| FD07-D10T7 | e3 ubiquitin-protein ligase at1g12760 | *Solanum tuberosum* | XP_006356611 | 6.61E-65 |
| FD07-D11T7 | lysine histidine transporter 1-like | *Cucumis sativus* | XP_004159807 | 6.37E-19 |
| FD07-E04T7 | 40s ribosomal protein s13-like | *Nicotiana tomentosiformis* | XP_009596116 | 7.15E-44 |
| FD07-E06T7 | hypothetical protein. partial | *Olea europaea* | AFP49328 | 4.22E-06 |
| FD07-E10T7 | chlorophyll a-b binding protein chloroplastic-like | *Nicotiana sylvestris* | XP_009783947 | 4.55E-31 |
| FD07-F06T7 | 60s ribosomal protein l8-like | *Aegiceras corniculatum* | AEX91926 | 1.19E-59 |
| FD07-H02T7 | gdsl esterase lipase at2g04570-like | *Coffea canephora* | CDP05713 | 3.97E-88 |
| FD07-H05T7 | protein sensitive to proton rhizotoxicity 1 | *Theobroma cacao* | XP_007020278 | 1.98E-17 |
| FD07-H11T7 | 20 kda chloroplastic-like | *Coffea canephora* | CDP03050 | 4.69E-51 |
| FD07-F09T7 | calmodulin-like protein 1 | *Coffea canephora* | CDO97169 | 2.19E-19 |
| FD07-F12T7 | stem-specific protein tsjt1-like | *Glycine max* | ACU23732 | 1.09E-49 |
| FD08-A02T7 | plasma membrane intrinsic protein | *Morus alba var. multicaulis* | AHZ08398 | 1.30E-65 |
| FD08-A08T7 | protein tic 20- chloroplastic | *Theobroma cacao* | XP_007020500 | 5.72E-32 |
| FD08-A12T7 | somatic embryogenesis receptor kinase 1-like | *Ricinus communis* | XP_002530200 | 2.09E-40 |
| FD08-B01T7 | secretory peroxidase | *Camellia oleifera* | ACT21094 | 3.62E-32 |
| FD08-B10T7 | macpf domain-containing protein cad1-like | *Coffea canephora* | CDP19447 | 8.31E-25 |
| FD08-B12T7 | auxin signaling f-box 2 | *Nicotiana tomentosiformis* | XP_009604230 | 8.84E-72 |
| FD08-C03T7 | chain a family protein | *Populus trichocarpa* | XP_002307245 | 9.89E-31 |
| FD08-C09T7 | leucine-rich repeat family protein | *Eucalyptus grandis* | XP_010028214 | 2.12E-07 |
| FD08-D02T7 | inactive beta-amylase 9-like | *Malus domestica* | XP_008340845 | 1.26E-34 |
| FD08-D03T7 | ubiquitin system component cue | *Erythranthe guttata* | EYU34978 | 1.36E-41 |
| FD08-D06T7 | photosystem ii reaction center w chloroplastic-like | *Lotus japonicus* | AFK47170 | 1.27E-24 |
| FD08-E04T7 | chlorophyll a-b binding protein chloroplastic-like | *Solanum lycopersicum* | XP_004246325 | 2.26E-133 |
| FD08-E10T7 | thioredoxin family protein | *Solanum lycopersicum* | XP_004230291 | 4.16E-78 |
| FD08-F01T7 | heat shock protein 70 | *Sandersonia aurantiaca* | AAL85887 | 3.41E-93 |
| FD08-F04T7 | ribulose- -bisphosphate carboxylase oxygenase activase | *Olea europaea* | ABS72022 | 2.25E-91 |
| FD08-G04T7 | protein thylakoid chloroplastic-like | *Erythranthe guttata* | EYU26097 | 3.14E-22 |
| FD08-G11T7 | polyubiquitin | *Bipolaris oryzae* | XP_007693173 | 9.31E-98 |
| FD08-H03T7 | gdp-mannose -epimerase 1-like | *Citrus sinensis* | XP_006486731 | 2.10E-35 |
| FD08-H06T7 | **major allergen pru ar 1-like Pathogenesis related protein 10** | *Eucalyptus grandis* | KCW59979 | 4.92E-08 |
| FD09-A03T7 | glyceraldehyde-3-phosphate dehydrogenase chloroplastic | *Olea europaea* | ABS72003 | 3.44E-50 |
| FD09-A05T7 | protein lhcp translocation defect | *Populus trichocarpa* | XP_006370666 | 1.96E-30 |
| FD09-A11T7 | protein dehydration-induced 19 homolog 3-like | *Citrus clementina* | XP_006433494 | 2.56E-33 |
| FD09-B03T7 | beta-amylase chloroplastic-like | *Coffea canephora* | CDP20299 | 1.94E-162 |
| FD09-B07T7 | tbc domain-containing family protein | *Erythranthe guttata* | EYU46428 | 9.85E-40 |
| FD09-C05T7 | polyphenol oxidase | *Coffea canephora* | CDP06099 | 8.08E-79 |
| FD09-C06T7 | cysteine proteinase inhibitor | *Knorringia sibirica* | ADD69946 | 3.57E-11 |
| FD09-D10T7 | 60s ribosomal protein l21-1-like | *Musa acuminata subsp. malaccensis* | XP_009397901 | 1.03E-70 |
| FD09-E01T7 | protein curvature thylakoid chloroplastic | *Citrus sinensis* | KDO86377 | 3.81E-17 |
| FD09-E02T7 | stem-specific protein tsjt1-like | *Avicennia marina* | AAK50814 | 2.46E-30 |
| FD09-E11T7 | galactinol synthase 1-like | *Ajuga reptans* | Q9XGN4 | 7.51E-19 |
| FD09-F04T7 | chain a family protein | *Catharanthus roseus* | AAU95203 | 5.27E-30 |
| FD09-G07T7 | zinc finger and btb domain-containing protein 11 isoform 1 | *Malus domestica* | XP_008339069 | 4.65E-43 |
| FD09-G09T7 | sec14 cytosolic factor-like | *Jatropha curcas* | KDP39300 | 1.53E-15 |
| FD10-A06T7 | probable rhamnose biosynthetic enzyme 1 | *Vitis vinifera* | CAN79484 | 3.57E-88 |
| FD10-C04T7 | probable lrr receptor-like serine threonine-protein kinase at2g23950 | *Coffea canephora* | CDP02874 | 3.94E-41 |
| FD10-C06T7 | outer envelope pore protein chloroplastic | *Nicotiana tomentosiformis* | XP_009588682 | 2.04E-58 |
| FD10-C02T7 | acyl carrier protein mitochondrial-like | *Nicotiana tomentosiformis* | XP_009598879 | 3.91E-52 |
| FD10-D06T7 | gras family transcription factor isoform 1 | *Solanum lycopersicum* | NP_001234310 | 1.22E-62 |
| FD10-D12T7 | formate dehydrogenase | *Ricinus communis* | XP_002517338 | 5.84E-157 |
| FD10-E02T7 | root r-b1-like | *Vitis vinifera* | XP_002275749 | 8.37E-67 |
| FD10-E03T7 | magnesium-protoporphyrin ix monomethyl ester | *Medicago truncatula* | ACJ85380 | 9.82E-98 |
| FD10-E05T7 | 50s ribosomal protein chloroplastic-like | *Nicotiana sylvestris* | XP_009797179 | 5.10E-38 |
| FD10-E06T7 | 29 kda ribonucleoprotein chloroplastic | *Amborella trichopoda* | XP_006846809 | 9.39E-39 |
| FD10-E07T7 | molybdate-anion transporter-like | *Erythranthe guttata* | EYU45290 | 1.11E-53 |
| FD10-F03T7 | serine threonine-protein kinase blus1 | *Erythranthe guttata* | EYU44754 | 1.32E-28 |
| FD10-F08T7 | 40s ribosomal protein s15a-1 | *Cicer arietinum* | XP_004503959 | 9.16E-73 |
| FD10-G03T7 | e3 ubiquitin protein ligase drip2-like | *Erythranthe guttata* | EYU46501 | 2.11E-12 |
| FD10-G04T7 | hypothetical protein CICLE_v10022209mg | *Citrus clementina* | XP_006443627 | 1.69E-16 |
| FD10-G07T7 | 40s ribosomal protein s7-like | *Erythranthe guttata* | EYU34408 | 6.14E-65 |
| FD10-G10T7 | nad h-quinone oxidoreductase subunit l | *Nicotiana sylvestris* | XP_009787590 | 3.44E-26 |
| FD10-H01T7 | abc transporter f family member 5-like | *Erythranthe guttata* | EYU31097 | 6.59E-63 |
| FD10-H11T7 | catalase | *Prunus persica* | CAB56850 | 2.18E-72 |
| FD10-H06T7 | polyubiquitin | *Nymphaea hybrid cultivar* | BAJ61942 | 6.49E-76 |
| FD11-A06T7 | lag1 longevity assurance homolog 2-like | *Erythranthe guttata* | EYU42675 | 9.78E-18 |
| FD11-A12T7 | serine hydroxymethyltransferase | *Cucumis sativus* | ABK55697 | 1.49E-52 |
| FD11-B04T7 | cinnamoyl- reductase 1-like | *Nicotiana sylvestris* | XP_009802199 | 2.98E-05 |
| FD11-B11T7 | cbs domain-containing protein mitochondrial | *Citrus sinensis* | KDO47216 | 8.16E-55 |
| FD11-C04T7 | ferredoxin- chloroplastic-like | *Nicotiana sylvestris* | XP_009796636 | 1.53E-41 |
| FD11-C05T7 | glyceraldehyde-3-phosphate dehydrogenase | *Scoparia dulcis* | AEO45783 | 4.35E-110 |
| FD12-C08T7 | photosystem ii core complex proteins chloroplastic-like | *Vitis vinifera* | XP_002285325 | 1.90E-31 |
| FD11-C11T7 | sedoheptulose- - chloroplastic-like | *Nicotiana sylvestris* | XP_009776701 | 4.20E-13 |
| FD11-D08T7 | translation factor sui1 | *Catharanthus roseus* | ADK79108 | 1.26E-08 |
| FD11-E10T7 | calcium-binding allergen ole e 8-like | *Solanum lycopersicum* | XP_004235822 | 2.47E-23 |
| FD11-E11T7 | universal stress protein a-like protein | *Vitis vinifera* | XP_002275863 | 4.28E-52 |
| FD11-F03T7 | bi1-like protein | *Erythranthe guttata* | EYU43851 | 1.22E-41 |
| FD11-F06T7 | hypothetical protein MIMGU-mgv1a017106mg | *Erythranthe guttata* | EYU31060 | 1.59E-12 |
| FD11-F09T7 | dna-binding family protein | *Nicotiana tomentosiformis* | XP_009613547 | 7.92E-15 |
| FD11-G08T7 | histone | *Zea mays* | ACG48841 | 9.94E-41 |
| FD11-G12T7 | cop9 signalosome complex subunit 2 | *Pyrus x bretschneideri* | XP_009371398 | 8.92E-145 |
| FD11-H02T7 | chlorophyll a b binding | *Citrus sinensis* | XP_006474704 | 5.59E-72 |
| FD11-H03T7 | hmg1 2-like | *Nicotiana sylvestris* | XP_009794035 | 1.81E-28 |
| FD11-H09T7 | probable ribose-5-phosphate isomerase chloroplastic | *Erythranthe guttata* | EYU36777 | 1.93E-38 |
| FD12-A01T7 | aldo-keto reductase family 4 member c9-like | *Citrus clementina* | XP_006429177 | 1.04E-49 |
| FD12-B06T7 | protein yls9-like | *Erythranthe guttata* | EYU18128 | 6.65E-39 |
| FD12-B09T7 | lysine-specific demethylase jmj25 | *Nicotiana sylvestris* | XP_009788374 | 3.05E-53 |
| FD12-B11T7 | unknown | *Glycine max* | ABK93304 | **7.88E-03** |
| FD12-C02T7 | PREDICTED: uncharacterized protein LOC104246313 | *Nicotiana sylvestris* | XP_009800415 | 2.38E-55 |
| FD12-C07T7 | zinc finger a20 and an1 domain-containing stress-associated protein 8-like | *Erythranthe guttata* | EYU31356 | 3.60E-16 |
| FD12-C10T7 | 60s ribosomal protein l28-1-like | *Nicotiana sylvestris* | XP_009795585 | 3.38E-83 |
| FD12-C12T7 | photosystem ii repair protein psb27- chloroplastic-like | *Phaseolus vulgaris* | XP_007149873 | 6.89E-48 |
| FD12-D02T7 | acyl- -binding protein | *Nicotiana tomentosiformis* | XP_009609336 | 5.53E-30 |
| FD12-D05T7 | -oxoglutarate 3-dioxygenase-like | *Nicotiana tomentosiformis* | XP_009608170 | 3.55E-33 |
| FD12-D10T7 | auxin-repressed kda | *Genlisea aurea* | EPS67742 | 3.72E-21 |
| FD12-E01T7 | casein kinase ii subunit beta | *Erythranthe guttata* | EYU17867 | 1.37E-47 |
| FD12-E10T7 | proteasome subunit alpha type-6 | *Genlisea aurea* | EPS63192 | 7.87E-15 |
| FD12-F03T7 | chlorophyll a-b binding protein chloroplastic-like | *Vitis vinifera* | XP_002264295 | 5.94E-54 |
| FD12-F04T7 | dna-damage-repair toleration protein drt100-like | *Solanum lycopersicum* | XP_004229610 | 1.75E-11 |
| FD12-G05T7 | beta-amyrin synthase | *Pyrus x bretschneideri* | XP_009347736 | 2.17E-25 |
| FD12-G09T7 | fiber protein fb34 | *Jatropha curcas* | KDP27023 | 5.43E-92 |
| FD12-H11T7 | small g protein signaling modulator 2-like | *Coffea canephora* | CDP05011 | 3.20E-17 |
| FD13-A01T7 | tetraspanin-8-like | *Nicotiana tomentosiformis* | XP_009595213 | 5.27E-27 |
| FD13-A06T7 | fructose-bisphosphate cytoplasmic isozyme 1 | *Solanum tuberosum* | AAQ90153 | 7.60E-41 |
| FD13-B08T7 | selenium-binding protein 2 | *Coffea canephora* | CDP15102 | 9.09E-76 |
| FD13-C11T7 | ubiquitin-conjugating enzyme e2-17 kda-like | *Setaria italica* | XP_004965488 | 2.06E-36 |
| FD13-D08T7 | dnaj homolog subfamily b member 11-like | *Solanum tuberosum* | XP_006349203 | 5.26E-39 |
| FD13-E03T7 | thaumatin-like protein | *Prunus mume* | XP_008238818 | 3.50E-06 |
| FD13-G09T7 | 60s ribosomal protein l11-1-like | *Nicotiana tomentosiformis* | XP_009629723 | 2.20E-93 |
| FD13-H01T7 | chloroplast stem-loop binding protein of 41 kda chloroplastic | *Nicotiana sylvestris* | XP_009780849 | 4.11E-80 |
| FD13-H11T7 | gdsl esterase lipase at5g33370-like | *Pyrus x bretschneideri* | XP_009379726 | 2.33E-41 |
| FD14-H01T7 | chlorophyll a-b binding protein chloroplastic-like | *Vitis vinifera* | CBI23318 | 2.74E-47 |
| FD14-A05T7 | zeaxanthin epoxidase | *Scutellaria baicalensis* | AGN03858 | 1.45E-103 |
| FD14-A07T7 | xanthine dehydrogenase 1-like | *Humulus lupulus* | ABS17591 | 3.00E-28 |
| FD14-A08T7 | protein translation factor sui1 homolog | *Phoenix dactylifera* | XP_008784556 | 1.34E-12 |
| FD14-A09T7 | ethylene-responsive transcription factor rap2-12-like | *Vitis vinifera* | XP_002267008 | 2.42E-50 |
| FD14-B06T7 | zinc protease pqql | *Nicotiana tomentosiformis* | XP_009617430 | 1.57E-47 |
| FD14-C11T7 | geranylgeranyl reductase | *Morus notabilis* | XP_010091526 | 7.19E-07 |
| FD14-D04T7 | gem-like protein 4-like | *Erythranthe guttata* | EYU22300 | 1.37E-37 |
| FD14-E01T7 | achain crystal structure of perakine founder member of a novel akr subfamily with unique conformational changes during nadph binding | *Olea europaea* | ABS72001 | 4.39E-27 |
| FD14-E05T7 | probable protein phosphatase 2c 25 | *Vitis vinifera* | CAN79916 | 7.11E-44 |
| FD14-F11T7 | homeodomain-like superfamily isoform partial | *Theobroma cacao* | XP_007042352 | 7.41E-31 |
| FD14-F12T7 | abscisic acid-responsive isoform 1 | *Theobroma cacao* | XP_007017782 | 3.75E-26 |
| FD14-G02T7 | dual specificity protein phosphatase 1-like | *Eucalyptus grandis* | KCW84288 | 2.34E-17 |
| FD14-H08T7 | transmembrane ascorbate ferrireductase 1-like | *Jatropha curcas* | KDP22285 | 5.62E-44 |
| FD-C6 | plastocyanin chloroplast | *Solanum lycopersicum* | XP_004238398 | 2.19E-43 |
| FD-C14 | nadp-dependent d-sorbitol-6-phosphate dehydrogenase-like | *Eucalyptus grandis* | KCW64076 | 9.46E-22 |
| FD-C16 | **salicylic acid-binding protein 2-like** | *Solanum lycopersicum* | **XP_004234906** | **5.82E-17** |
| FD-C31 | chlorophyll a-b binding protein chloroplastic | *Ricinus communis* | XP_002531690 | 1.44E-139 |
| FD-C37 | phosphate-responsive 1 family protein | *Coffea canephora* | CDP01092 | 5.47E-62 |
| FD-C44 | photosystem ii 10 kda chloroplastic | *Vitis vinifera* | XP_002271791 | 7.86E-47 |
| FD-C45 | u-box domain-containing protein 72 | *Vitis vinifera* | CAN74845 | 9.19E-33 |
| FD-C48 | chlorophyll a-b binding protein chloroplastic-like | *Musa acuminata subsp. malaccensis* | XP_009389379 | 7.61E-149 |
| FD-C50 | photosystem ii 10 kda chloroplastic | *Nicotiana sylvestris* | XP_009774551 | 6.53E-59 |
| FD-C51 | polyubiquitin-like protein | *Citrus maxima* | ADC29516 | 8.57E-53 |
| FD-C52 | cysteine protease | *Erythranthe guttata* | EYU28328 | 1.30E-83 |
| FD-C55 | photosystem i reaction center subunit chloroplastic | *Phillyrea latifolia* | CAK18849 | 8.40E-65 |
| FD-C60 | chlorophyll a-b binding protein chloroplastic-like | *Prunus mume* | XP_008229753 | 1.14E-80 |
| FD-C65 | **dirigent protein 21-like** | *Erythranthe guttata* | **EYU31570** | 3.70E-37 |
| FD-C67 | chlorophyll a-b binding protein chloroplastic-like | *Sorghum bicolor* | XP_002455901 | 1.85E-140 |
| FD-C69 | saga-associated factor 29 homolog isoform x2 | *Coffea canephora* | CDP16615 | 5.82E-37 |
| FD-C68 | photosystem i reaction center subunit chloroplastic-like | *Erythranthe guttata* | EYU41499 | 8.06E-68 |
| FD-C73 | chloroplast import receptor p36 family protein | *Citrus sinensis* | KDO77700 | 9.21E-90 |
| FD-C77 | polyubiquitin | *Medicago sativa* | AAZ32851 | 6.56E-104 |
| FD-C81 | metallothionein-like protein | *Plantago major* | CAH59436 | 2.11E-07 |
| FD-C82 | dehydrin | *Paraboea crassifolia* | AAF01465 | 8.54E-10 |
| FD-C86 | sec14 cytosolic factor family protein phosphoglyceride transfer family protein | *Theobroma cacao* | XP_007017939 | 1.71E-37 |
| FD-C94 | superoxide dismutase | *Solanum nigrum* | ACJ73904 | 1.17E-22 |
| FD-C97 | selt-like protein | *Erythranthe guttata* | EYU41911 | 1.82E-13 |
| FD-C98 | kunitz family trypsin and protease inhibitor | *Fragaria vesca subsp. vesca* | XP_004299457 | 7.95E-21 |
| FD-C99 | metallothionein-like protein | *Eutrema salsugineum* | XP_006406981 | 2.64E-07 |
| FD-C112 | oxygen-evolving enhancer protein 3- chloroplastic | *Coffea canephora* | CDO99700 | 4.37E-27 |
| FD-C114 | membrane family protein | *Nicotiana sylvestris* | XP_009798442 | 4.22E-30 |
| FD-C120 | mitochondrial | *Solanum tuberosum* | XP_006348138 | 8.63E-84 |
| FD-C125 | alpha beta hydrolase fold superfamily | *Catharanthus roseus* | AAU95203 | 3.85E-66 |
| FD-C129 | thaumatin-like protein | *Olea europaea* | E3SU11 | 9.31E-49 |
| FD-C141 | ribulose bisphosphate carboxylase small chloroplastic | *Erythranthe guttata* | EYU18843 | 9.57E-21 |
| FD-C147 | aldolase-type tim barrel family protein isoform 1 | *Erythranthe guttata* | EYU38211 | 5.06E-66 |
| FD-C154 | s-adenosylmethionine synthase 2 | *Genlisea aurea* | EPS60608 | 4.37E-95 |
| FD-C156 | metallothionein 1 | *Olea europaea* | AFP49330 | 5.50E-15 |
| FD-C163 | salt tolerance-related protein | *Olea europaea* | ABS72020 | 4.80E-62 |
| FD-C175 | unnamed protein product | *Vitis vinifera* | CBI40860 | 7.17E-43 |
| FD-C188 | peptidyl-prolyl cis-trans partial | *Betula pendula* | CAC84116 | 6.46E-49 |
| FD-C194 | chlorophyll a-b binding protein chloroplastic-like | *Solanum lycopersicum* | XP_004246325 | 2.99E-134 |
| FD-C203 | beta- glucosidase partial protein | *Olea europaea* | AAL93619 | 1.78E-111 |
| FD-C207 | auxin-binding protein abp19a-like | *Erythranthe guttata* | EYU25861 | 1.53E-52 |
