# Supplementary material for: Systemic responses in a tolerant olive (Olea europaea L.) cultivar upon root colonization by the vascular pathogen Verticillium dahliae
Source: Front Microbiol. 2015 Sep 16;6:928. doi: 10.3389/fmicb.2015.00928 (PMC4584997; doi:10.3389/fmicb.2015.00928)
Supplement: Supplementary file 3 [file Table3.DOC]

| **Table S3**. List of contigs and their corresponding contiguous/overlapping ESTs. The EST Sequence Name refers to the codes (User-IDs) found within the FU cDNA library. FU means Frantoio aerial tissues repressed gene and FU-C indicates Frantoio aerial tissues identified as part of a contig. T7 refers to the forward T7 universal primers used for sequencing. | |
| --- | --- |
| **Contig names used in this study** | **EST sequences names (User-IDs) as found in dbEST/dbGSS/dbSTS databases** |
| FU-C1 | FU01-A01T7, FU12-C07T7 |
| FU-C10 | FU01-B11T7, FU09-B11T7 |
| FU-C11 | FU01-C01T7, FU01-E05T7, FU06-A06T7, FU03-A10T7 |
| FU-C15 | FU01-C08T7, FU10-G04T7 |
| FU-C17 | FU01-C11T7, FU06-H03T7, FU04-G05T7, FU01-H10T7 |
| FU-C24 | FU01-E01T7, FU09-F08T7 |
| FU-C25 | FU14-G10T7, FU01-E04T7 |
| FU-C32 | FU01-F04T7, FU08-H10T7 |
| FU-C33 | FU01-F05T7, FU10-E11T7 |
| FU-C35 | FU01-F09T7, FU06-E10T7 |
| FU-C36 | FU03-A02T7, FU01-F10T7, FU12-C11T7 |
| FU-C40 | FU01-G06T7, FU09-G08T7 |
| FU-C44 | FU01-H04T7, FU03-H01T7 |
| FU-C47 | FU02-A02T7, FU14-C06T7 |
| FU-C50 | FU02-A06T7, FU04-A03T7, FU07-E08T7, FU05-H02T7 |
| FU-C58 | FU02-E08T7, FU02-C03T7 |
| FU-C59 | FU02-C04T7, FU13-A01T7 |
| FU-C64 | FU02-D04T7, FU05-C09T7 |
| FU-C67 | FU02-D10T7, FU06-G08T7 |
| FU-C68 | FU02-D11T7, FU12-E03T7 |
| FU-C72 | FU02-E06T7, FU14-E10T7 |
| FU-C75 | FU02-F04T7, FU07-D10T7 |
| FU-C79 | FU02-G01T7, FU03-E12T7 |
| FU-C91 | FU03-B02T7, FU11-F06T7 |
| FU-C92 | FU03-B04T7, FU03-B09T7 |
| FU-C102 | FU10-F07T7, FU03-D04T7, FU04-H12T7, FU05-G02T7 |
| FU-C104 | FU03-D08T7, FU13-F06T7 |
| FU-C108 | FU03-E06T7, FU08-A06T7 |
| FU-C109 | FU08-H05T7, FU03-E10T7, FU09-A10T7 |
| FU-C111 | FU03-F01T7, FU14-G07T7 |
| FU-C113 | FU03-F07T7, FU09-F02T7 |
| FU-C114 | FU03-F10T7, FU08-D12T7 |
| FU-C119 | FU03-G07T7, FU10-B08T7 |
| FU-C126 | FU04-D07T7, FU04-F11T7, FU07-C04T7 |
| FU-C129 | FU04-E08T7, FU14-A07T7 |
| FU-C130 | FU04-H05T7, FU09-F01T7 |
| FU-C133 | FU04-B02T7, FU07-B05T7, FU12-C06T7 |
| FU-C138 | FU04-E06T7, FU06-G12T7 |
| FU-C144 | FU04-G12T7, FU05-D11T7 |
| FU-C145 | FU04-D02T7, FU07-F12T7, FU10-G11T7 |
| FU-C146 | FU04-D03T7, FU07-F08T7 |
| FU-C147 | FU04-F07T7, FU06-E12T7 |
| FU-C150 | FU04-F12T7, FU11-C07T7 |
| FU-C165 | FU07-D07T7, FU14-A05T7 |
| FU-C168 | FU06-D01T7, FU07-E05T7 |
| FU-C171 | FU11-E01T7, FU13-F05T7 |
| FU-C191 | FU08-D11T7, FU10-A02T7 |
| FU-C195 | FU08-G07T7, FU12-B10T7 |
| FU-C202 | FU11-F11T7, FU06-G03T7 |
| FU-C203 | FU09-C06T7, FU06-H09T7 |
| FU-C224 | FU10-D12T7, FU10-G09T7, FU05-C04T7, FU05-G01T7 |
| FU-C228 | FU10-F11T7, FU11-C03T7 |
| FU-C235 | FU11-C09T7, FU13-F01T7 |
| FU-C243 | FU12-A08T7, FU13-D03 |
| FU-C256 | FU05-A09T7, FU05-B12T7 |
| FU-C258 | FU06-C12T7, FU05-D09T7 |
| FU-C259 | FU06-D02T7, FU06-D09T7 |
