# Supplementary material for: Systemic responses in a tolerant olive (Olea europaea L.) cultivar upon root colonization by the vascular pathogen Verticillium dahliae
Source: Front Microbiol. 2015 Sep 16;6:928. doi: 10.3389/fmicb.2015.00928 (PMC4584997; doi:10.3389/fmicb.2015.00928)
Supplement: Supplementary file 4 [file Table4.DOC]

| **Table S4**. List of contigs and their corresponding contiguous/overlapping ESTs. The EST Sequence Name refers to the codes (User-IDs) found within the FD cDNA library. FD means Frantoio aerial tissues repressed gene and FD-C indicates Frantoio aerial tissues identified as part of a contig. T7 refers to the forward T7 universal primers used for sequencing. | |
| --- | --- |
| **Contig names used in this study** | **EST sequences names (User-IDs) as found in dbEST/dbGSS/dbSTS databases** |
| FD-C3 | FD01-A06T7, FD09-H01T7, FD14-F05T7 |
| FD-C6 | FD01-E02T7, FD03-A02T7 |
| FD-C14 | FD08-B02T7, FD01-A08T7 |
| FD-C16 | FD04-A02T7, FD09-D09T7, FD14-E10T7, FD12-F09T7, FD14-H03T7, FD01-F02T7 |
| FD-C23 | FD03-G10T7, FD12-H02T7, FD12-H07T7, FD05-C12T7, FD01-D02T7, FD09-B10T7, FD14-C02T7,FD12-F10T7, FD14-D12T7, FD09-H10T7,FD02-F09T7, FD06-C01T7, FD13-G02T7, FD14-E12T7 |
| FD-C31 | FD09-A07T7, FD09-C03T7, FD11-H12T7, FD02-C10T7, FD13-H10T7 |
| FD-C33 | FD01-A12T7, FD06-H10T7, FD13-H02T7, FD02-E07T7 |
| FD-C37 | FD01-F12T7, FD06-H05T7, FD05-F12T7 |
| FD-C44 | FD05-H11T7, FD02-G10T7,FD02-C01T7, FD14-C01T7, FD01-D09T7, FD10-D04T7 |
| FD-C45 | FD06-A09T7, FD01-F05T7 |
| FD-C48 | FD14-A11T7, FD02-F12T7, FD01-G12T7 |
| FD-C50 | FD01-D07T7, FD10-H10T7, FD11-D07T7, FD13-D01T7, FD13-A08T7, FD01-F08T7 |
| FD-C51 | FD02-G04T7, FD01-B05T7 |
| FD-C52 | FD04-H11T7, FD01-H11T7, FD06-F08T7 |
| FD-C55 | FD01-D10T7, FD03-A03T7 |
| FD-C56 | FD07-B06T7, FD01-E09T7 |
| FD-C57 | FD01-C05T7, FD04-E05T7, FD04-E01T7 |
| FD-C60 | FD02-E04T7, FD11-B07T7 |
| FD-C65 | FD03-H03T7, FD02-G11T7 |
| FD-C67 | FD02-E06T7, FD13-B04T7, FD02-D11T7, FD05-F03T7 |
| FD-C68 | FD02-C04T7, FD03-H08T7, FD12-H10T7 |
| FD-C69 | FD02-F10T7, FD10-C05T7 |
| FD-C73 | FD02-B04T7,FD07-D04T7, FD09-D01T7 |
| FD-C77 | FD02-A11T7, FD13-H06T7, FD03-C01T7, FD03-A05T7, FD09-A04T7, FD06-E10T7, FD13-D02T7, FD14-B02T7 |
| FD-C81 | FD09-D08T7, FD02-G06T7 |
| FD-C82 | FD02-H07T7, FD06-A11T7, FD05-F11T7 |
| FD-C86 | FD02-H08T7, FD11-E09T7 |
| FD-C94 | FD02-B07T7, FD08-G08T7 |
| FD-C97 | FD03-G05T7, FD11-A10T7 |
| FD-C98 | FD03-E05T7, FD06-C05T7, FD09-C07T7 |
| FD-C99 | FD12-A12T7, FD03-A11T7, FD13-B09T7, FD05-G01T7 |
| FD-C103 | FD03-H09T7, FD03-F03T7 |
| FD-C112 | FD03-E03T7, FD11-H07T7 |
| FD-C114 | FD03-E02T7, FD04-E07T7 |
| FD-C120 | FD04-B12T7, FD03-D02T7 |
| FD-C125 | FD03-B11T7, FD12-E08T7 |
| FD-C128 | FD03-C03T7, FD04-A09T7 |
| FD-C129 | FD06-C10T7, FD04-B11T7 |
| FD-C133 | FD06-G09T7, FD14-A10T7 |
| FD-C141 | FD11-C07T7, FD06-G08T7, FD07-E01T7, FD09-G10T7 |
| FD-C147 | FD07-G08T7, FD12-H05T7 |
| FD-C153a | FD12-E04T7, FD12-G04T7, FD11-G02T7 |
| FD-C153b | FD07-D06T7, FD10-B10T7 |
| FD-C154 | FD07-A04T7, FD12-A10T7 |
| FD-C156 | FD07-H09T7, FD14-G03T7 |
| FD-C163 | FD07-B03T7, FD09-F05T7, FD12-E12T7 |
| FD-C169 | FD09-H09T7, FD08-G01T7 |
| FD-C175 | FD08-C08T7, FD14-C06T7 |
| FD-C188 | FD09-H04T7, FD13-E09T7 |
| FD-C194 | FD10-E12T7, FD12-E11T7 |
| FD-C203 | FD05-G10T7, FD10-G12T7 |
| FD-C207 | FD11-D03T7, FD11-A08T7 |
