# Supplementary material for: Systemic responses in a tolerant olive (Olea europaea L.) cultivar upon root colonization by the vascular pathogen Verticillium dahliae
Source: Front Microbiol. 2015 Sep 16;6:928. doi: 10.3389/fmicb.2015.00928 (PMC4584997; doi:10.3389/fmicb.2015.00928)
Supplement: Supplementary file 5 [file Table5.DOC]

| **Table S5.** Means and standard deviations of relative expression genes at 2 and 10 days after *Verticillium dahliae* inoculation in three different olive cultivars. | | | | | | | | | | | | | | | |  |
| --- | --- | --- | --- | --- | --- | --- | --- | --- | --- | --- | --- | --- | --- | --- | --- | --- |
|  | **CHANGLOT** | | | | | | **FRANTOIO** | | | | | **PICUAL** | | | |  |
| **ACO** | | **Rep 1** | **Rep 2** | **Means** | **STD** |  | **Rep 1** | **Rep 2** | **Rep 3** | **Means** | **STD** | **Rep 1** | **Rep 2** | **Means** | **STD** |  |
| **T2 P1** | | -0.855 | -0.860 | -0.858 | 0.004 |  | 2.325 | 2.095 | 2.078 | 2.166 | 0.138 | -0.495 | -0.358 | -0.427 | 0.097 |  |
| **T2 P2** | | -1.203 | -1.233 | -1.218 | 0.021 |  | 1.340 | 1.365 | 1.308 | 1.338 | 0.028 | -0.708 | -0.795 | -0.752 | 0.061 |  |
| **T10 P1** | | 0.207 | 0.398 | 0.303 | 0.136 |  | -1.613 | -1.615 | -1.542 | -1.590 | 0.042 | 1.492 | 1.618 | 1.555 | 0.090 |  |
| **T10 P2** | | -0.397 | -0.205 | -0.301 | 0.136 |  | 0.370 | 0.597 | 0.538 | 0.502 | 0.118 | 1.828 | 2.088 | 1.958 | 0.184 |  |
| **DRR2** | | **Rep 1** | **Rep 2** | **Rep 3** | **Means** | **STD** | **Rep 1** | **Rep 2** | **Rep 3** | **Means** | **STD** | **Rep 1** | **Rep 2** | **Means** | **STD** |  |
| **T2 P1** | | -0.717 | -0.615 | -0.593 | -0.642 | 0.054 | -1.372 | -1.618 | -1.702 | -1.564 | 0.172 | 1.618 | 1.902 | 1.760 | 0.200 |  |
| **T2 P2** | | -1.328 | -1.637 | -1.440 | -1.468 | 0.127 | -0.762 | -0.885 | -0.422 | -0.689 | 0.240 | -0.268 | -0.215 | -0.242 | 0.038 |  |
| **T10 P1** | | -0.575 | -0.208 | -0.517 | -0.433 | 0.161 | -1.955 | -1.982 | -1.788 | -1.908 | 0.105 | 0.708 | 0.812 | 0.760 | 0.073 |  |
| **T10 P2** | | -0.238 | -0.212 | -0.487 | -0.312 | 0.124 | -1.312 | -1.328 | -1.242 | -1.294 | 0.046 | 1.025 | 1.437 | 1.231 | 0.291 |  |
| **ACL** | | **Rep 1** | **Rep 2** | **Means** | **STD** |  | **Rep 1** | **Rep 2** | **Means** | **STD** |  | **Rep 1** | **Rep 2** | **Means** | **STD** |  |
| **T2 P1** | | -2.505 | -2.465 | -2.485 | 0.028 |  | 4.373 | 4.155 | 4.264 | 0.154 |  | -2.272 | -1.802 | -2.037 | 0.332 |  |
| **T2 P2** | | -0.395 | -0.562 | -0.478 | 0.118 |  | 1.520 | 1.458 | 1.489 | 0.044 |  | 0.352 | 0.393 | 0.373 | 0.029 |  |
| **T10 P1** | | 2.085 | 2.182 | 2.133 | 0.068 |  | 0.013 | -0.502 | -0.244 | 0.364 |  | -1.295 | -0.818 | -1.057 | 0.337 |  |
| **T10 P2** | | -0.962 | -0.695 | -0.828 | 0.189 |  | -0.293 | -0.422 | -0.358 | 0.091 |  | -1.738 | -1.472 | -1.605 | 0.189 |  |
| **GRAS** | | **Rep 1** | **Rep 2** | **Means** | **STD** |  | **Rep 1** | **Rep 2** | **Means** | **STD** |  | **Rep 1** | **Rep 2** | **Means** | **STD** |  |
| **T2 P1** | | -1.518 | -1.195 | -1.357 | 0.229 |  | -0.185 | -0.632 | -0.408 | 0.316 |  | 0.838 | 0.938 | 0.888 | 0.071 |  |
| **T2 P2** | | -1.555 | -1.222 | -1.388 | 0.236 |  | -0.317 | -0.512 | -0.414 | 0.138 |  | 0.848 | 1.172 | 1.010 | 0.229 |  |
| **T10 P1** | | -0.702 | -0.655 | -0.678 | 0.033 |  | -0.120 | -0.195 | -0.157 | 0.053 |  | -1.228 | -1.278 | -1.253 | 0.035 |  |
| **T10 P2** | | -0.678 | -0.568 | -0.623 | 0.078 |  | -0.210 | -0.222 | -0.216 | 0.008 |  | -0.932 | -0.662 | -0.797 | 0.191 |  |
| ACO: 1-Aminocyclopropane-1-carboxylate oxidase. DRR2: Disease resistance-responsive family protein. ACL: Acetone cyanohydrin lyase. GRAS: Transcription factor GRAS1. T2: two days after inoculation. T10: ten days after inoculation. P: plant. Rep: replay. STD: standard deviation | | | | | | | | | | | | | | | | |
